# Classification of Acoustic Emission Signals from Failure Modes in Thermoplastic Composites Using Machine Learning

**DOI:** 10.3390/ma19204380

**Published:** 2026-10-19

**Authors:** Tanzila B. Minhaj, David Amoateng-Mensah, Manoj Rijal, Richard Dela Amevorku, Mannur J. Sundaresan

**Affiliations:** 1 Department of Mechanical Engineering, North Carolina A & T State University, 1601 E. Market Street, Greensboro, NC 27411, USA; damoatengmensah@aggies.ncat.edu (D.A.-M.); rdamevorku@aggies.ncat.edu (R.D.A.); mannur@ncat.edu (M.J.S.); 2 Independent Researcher, Greensboro, NC 27407, USA; rijalmanoj21@gmail.com

**Keywords:** acoustic emission, thermoplastic composite material, machine learning, deep learning, unsupervised classification

## Abstract

The evolution of the damage process in PAEK-type thermoplastic carbon fiber-reinforced polymer (CFRP) quasi-isotropic composite tensile specimens was investigated using acoustic emission (AE) monitoring under quasi-static loading to failure. Key objectives were to apply a deep learning (DL)-machine learning (ML) framework to the AE signals from tested specimens, identify the participating signal classes using unsupervised classification, and quantify their relative extent at different stages of the loading cycle. Unlike prior studies that rely on conventional AE features (i.e., amplitude, rise time, etc.), this work automated feature extraction directly from AE signals using a deep learning model. A 1D convolutional neural network (CNN) automatically extracted frequency and temporal features from AE events recorded from nominally identical specimens tested according to ASTM D3039. Principal component analysis reduced the high-dimensional feature space while retaining 85% of the variance, and spectral clustering identified four distinct classes validated by Davies–Bouldin index < 1.2 and Calinski–Harabasz index > 200 across both specimens. Fast Fourier transform analysis revealed that classes are primarily distinguished by frequency content. Low- and mid-frequency signals (<900 kHz), typically associated with delamination and matrix cracking in published literature, were relatively few. Two high-frequency classes emerged. One had mid-high frequency events (900–1600 kHz) with high amplitude, increasing steadily up to 70% of ultimate load, and the other very high-frequency events (>1600 kHz) with low amplitude, surging rapidly beyond 80% of ultimate load until failure. Cross-correlation analysis revealed minimal formation of groups of fiber breaks called the ‘multiplets’ characteristic, suggesting the ductile PAEK matrix suppresses clustered fiber damage. The proposed DL-ML framework exhibited promising results, with the prospect of eliminating dependence on subjective, test-setup-sensitive parameters and providing a reproducible methodology for damage classification in thermoplastic composites. Besides, automated feature extraction could improve the likelihood of early detection of real-life damage progression before final failure. As a potential mathematical model, the scope for further improvement includes that the performance of the current framework depends significantly on the preprocessing of the data (i.e., the right size of signal that has substantial information on damage modes of both short and long type with least noise, elimination of the amplitude parameter by choosing manual amplitude bin). Direct validation of frequency-to-damage mode assignments through synchronized in situ imaging remains necessary to confirm the damage modes.

## 1. Introduction

### 1.1. Background on Damage Identification in Thermoplastic CFRP Composites

Modern aircraft are mostly made of polymer-matrix composite materials. Newer matrix materials are being developed to overcome the drawbacks of the existing ones. The emergence of thermoplastic CFRP composite materials is comparatively recent and they are increasingly being considered due to their improved impact resistance, ease of manufacture, repairability, and recyclability [1].

However, the mechanical properties of the composites largely depend on the fiber orientations within the laminate [2,3] as well. Due to having minimal or no cross-linking, thermoplastic matrix materials are entirely heat-reversible up to a certain temperature, enabling reshaping, welding, and repair [1]. Highly cross-linked resins have limited molecular mobility, which results in brittle behavior, low failure strain, and poor impact resistance. On the other hand, the thermoplastic matrix materials demonstrate ductile failure and impact resistance. In addition, they can be recycled and reused, and they can have an unlimited life due to heat reversibility and better corrosion resistance. The physical entanglement of molecular chains within thermoplastic matrix materials strengthens the material while maintaining high plastic flow through the stretching and pulling of the chains. Thermoplastic CFRP composites are being actively considered for several aeronautical and automotive applications, and hence, it is important to monitor and detect damage in these material systems to prevent failures. Polyaryletherketones (PAEKs), used as the matrix in this research, have advanced among matrices due to their exceptional performance in environments requiring high endurance for chemical and thermal instabilities compared to other semicrystalline thermoplastics [4]. An extensive review on PAEK CFRPs by Veazey et al. [5] revealed their specific competitive strength and stiffness against certain metal alloys, also serving as a perfect candidate for both longer retained fibers and stronger fiber–matrix interfaces.

Structural health monitoring techniques can continuously track the accumulation of damage and improve the safety and durability of structural members in these applications. The types of damage and the rate of their accumulation in thermoplastic CFRP composites are different from those observed in thermoset CFRP composites and need to be better characterized.

To monitor damage evolution in thermoplastic CFRP composites, this study employs acoustic emission monitoring, a passive nondestructive technique that detects elastic waves generated by material damage [6,7]. This method offers real-time, in situ detection of damage events as they occur during loading, making it particularly valuable for understanding progressive failure in composite materials. Whenever damage occurs, the rapid localized release of strain energy generates transient elastic waves that propagate through the structure. As these stress waves travel, they cause alternating tension and compression movements that are recorded by surface-mounted sensors. In this research, the structure of interest is thin plates due to their numerous versatile applications in aircraft and automotive design. Thus, the type of elastic wave considered in the study was a plate wave, also known as a Lamb wave. When the AE energy propagates through a thin plate, the elastic wave, known as a Lamb wave, can have either an extensional or flexural mode [6].

The layup within a CFRP thermoplastic composite thin plate influences fracture modes and, ultimately, damage type. Cao et al. showed that the failure mode of a loaded plate depends on its boundary constraints and geometry. At the same time, the thickness-to-width ratio shifts the balance between bending-dominated tensile cracking and shear-dominated failure [8]. During quasi-static loading of CFRP thermoplastic composite tensile coupons, the following failure modes are expected: matrix cracking (within the matrix material), matrix splitting, delamination (between the layers), fiber breakage, and fiber-matrix debonding [7].

Since thermoplastic composites are an emerging material, researchers are investigating their characteristics. Research is still ongoing to properly quantify the frequency dispersion and attenuation of AE signals in this type of material. Modal acoustic emission analysis indicates that the matrix crack-type damage contains a dominant S_0_ mode, followed by an A_0_ mode [9]. On the other hand, delamination emits signals containing a dominant A_0_ mode with low frequency, and signals emitted from fiber-break events can be a combination of higher-order Lamb wave modes. It is theoretically evident that, with given modal characteristics, AE signals from delamination-type damage can have frequencies of around 100 to 250 kHz associated with high amplitude; AE signals emitted from matrix crack-type damage can contain around 250 to 650 kHz with medium peak amplitude, and a single fiber break having a minimal cross-sectional area while subjected to high load emits AE signals with above 700 kHz frequency [10,11,12,13,14] with low to medium peak amplitude.

### 1.2. Unsupervised Pattern Recognition in Classifying Damage Modes

Unlike supervised learning, unsupervised learning does not require labeled data, whereas ‘labeled data’ refers to two data sets in training data so that each example data x has one output label y [15]. The goal of supervised learning is to acquire the input–output mapping using different optimization techniques. However, unsupervised learning lets the model observe the data and classify it based on its own features rather than a user-defined label. Thus, unsupervised classification does not depend on tedious data training or optimization of the input–output mapping. Hence, this type of classification is not contingent on several hyperparameters such as batch size, learning rate, optimizer, etc., on which supervised classification results vary a lot. The challenge of this method is the lack of ground truth to compare. Clustering is a simple example of unsupervised learning, where the input data is partitioned into regions, so that ‘similar’ data points fall into the same region. A number of researchers have worked on unsupervised clustering approaches that utilize user-specified and provided input data of traditional AE parameters such as rise time, amplitude, peak frequency, absolute energy, etc., for partitioning AE waveforms from different failure modes [16,17,18,19]. Researchers have explored diverse effective parameters that can efficiently classify the damage signals. An AE-ML framework for power-based clustering was proposed by Muir et al. They partitioned the AE data after encoding it with a modified partial power scheme [20]. They found that the power distribution is more compact within a specific frequency range, above 70–85% of the ultimate tensile stress. Thus, they concluded that partial power is a strong and salient feature that distinguishes waveforms responsible for different damage modes. Spectral clustering, a type of clustering model, is effective for this type of AE signal data and consistently outperforms k-means clustering [21,22]. Unlike the traditional approach, this paper examines deep learning models for extracting data features to recognize patterns, then employs unsupervised learning to cluster them based on those features.

### 1.3. Background of Feature Extraction Techniques for Unsupervised Learning

Studying the damage source involves studying damage features. Teng et al. showed that fractal dimension is capable of tracking damage stage transitions in compressed coal, motivating complexity-based features [23]. Since the AE waveform contains features that depend on the damage source [12,16,24,25,26], studying the features of AE signals can be effective for understanding the nature of damage. However, researchers have found conventional AE features unreliable for assessing damage modes [19,27,28]. Those AE parameters are somewhat subjective because the value of such parameters is unduly affected by the experimental setup, such as the gain and threshold used for data acquisition, sensor type, and their location. Such distortions can render the identification of failure modes and prediction of the criticality of damage unreliable [7]. To address this problem, in the current approach, three off-the-shelf algorithms were concatenated. The three algorithms consisted of three 1D convolutional neural networks (CNNs) for automatic feature extraction followed by principal component analysis (PCA) for dimensionality reduction, and spectral clustering for classification. Instead of providing traditional AE parameters, the adaptation of these three algorithms enabled automated extraction of the most relevant features from the AE signals that differentiate the waveforms for different failure modes and then partition the data so that the signals with similar features are in the same group.

It is well known that polymeric composites, due to loading, generate an enormous number of AE signals, making manual analysis impossible. The recent AI revolution has emerged to resolve the situation and has offered to undertake the mammoth task in a short time. Harnessing deep learning model feature engineering, a subset of machine learning, has become a state-of-the-art approach. Thus, it has greater potential to be more reliable and less dependent on test setup, sensor choice, or location, etc.

Deep learning-based feature extraction has been shown to perform well on low-frequency signals [24]. Deep scattering is highly effective at extracting features from audio signals and electrocardiograms [25,26,29,30]. It uses the Morlet mother wavelet to extract frequency features. Speech recognition is a very similar and popular example of unsupervised learning, where features from signal spectrograms are extracted using the mel-frequency cepstral coefficients (MFCC) method [31], with preference given to lower frequency ranges [32]. Linear chirp transformation has been utilized in speech emotion-recognition automation [33,34,35] due to its advantageous properties [36]. The dynamic nature of the frequency of damage modes, ranging from 50 kHz to 3.5 MHz, along with the importance of the amplitude or energy associated with each frequency component, makes unsupervised learning of AE signals from damage in composites based on automatic feature extraction a challenging task.

This paper explores the novel application of the DL-ML model for studying damage modes responsible for thermoplastic CFRP composite specimen failure. The proposed DL-ML framework performed well on large volumes of AE data and extracted important features from signals with frequency components as low as 50 kHz and as high as 3 MHz. The first part of the model comprises three layers of 1D-CNN models for feature extraction, followed by principal component analysis for dimensionality reduction and feature transformation. In the second part, the waveforms are classified using an unsupervised learning model called spectral clustering. The fast Fourier transform (FFT) validated the most relevant feature extracted from the DL-ML framework. The tests on several specimens demonstrated the consistency of the findings and justified the functionality of the mathematical model. However, the paper provides results from two sensors of two test specimens. The nominally identical specimens were used because the objective of the current study was to develop a mathematical model that can cluster signal data independently, without predefined parameter sets, followed by investigation of the groups to gain insight into damage in the specimens. The strong relevance of the principal components to the features of the signals obtained from the 1D-CNN models suggests applying the model to substantively distinct specimens in the future and improving the robustness of the mathematical model.

## 2. Materials and Methods

### 2.1. Experimental Setup

**Figure 1:**
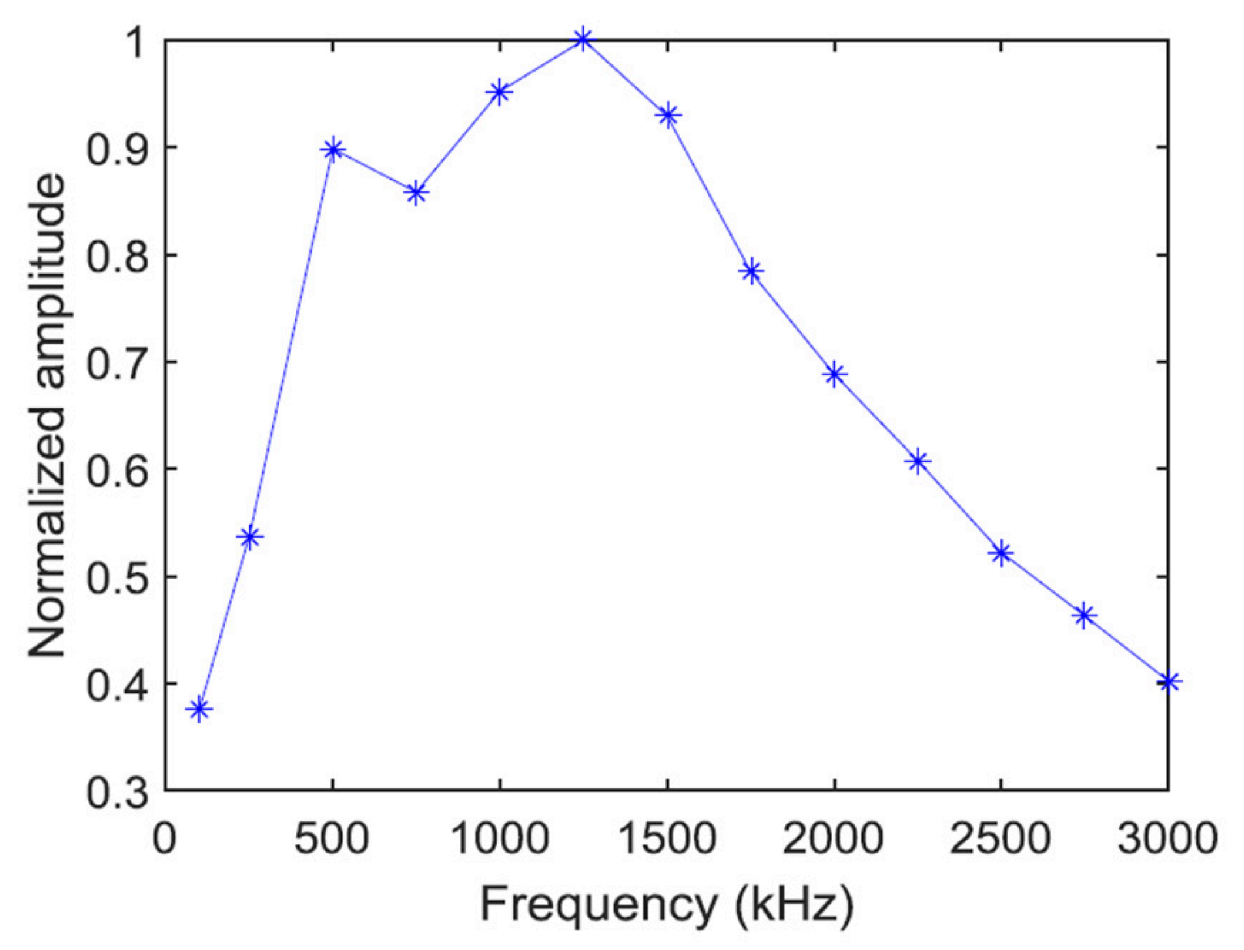
Frequency response of the bonded piezoelectric wafer sensor [38].

The results of this paper are based on acoustic emission signals collected by bonded piezoelectric wafer sensors developed in-house, which are designed to detect the in-plane strain resulting from propagating Lamb waves in thin plates [37]. Such sensors are shown to be advantageous in terms of wider frequency response and superior ability to quantify acoustic emission energy. The frequency response of the sensors was calibrated using Lamb wave pulses of different frequencies ranging from 100 kHz to 3 MHz [38], as illustrated in Figure 1 below.

#### 2.1.1. Specimen Preparation and Loading

**Figure 2:**
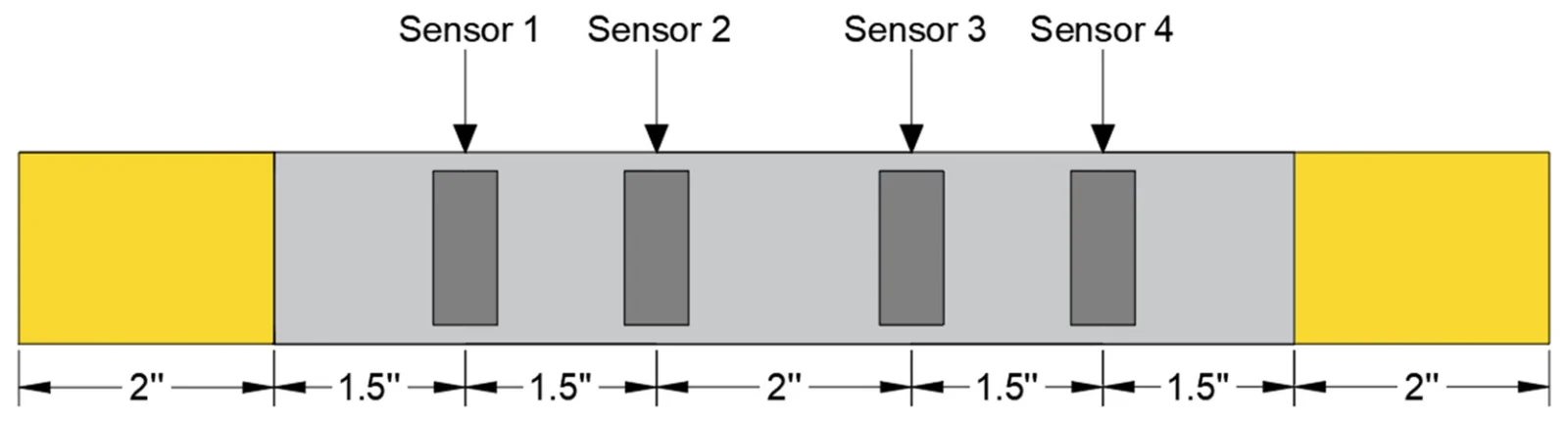
The test specimen [40].

Tensile specimens from PAEK thermoplastic CFRP composite were prepared in accordance with ASTM Standard D3039 [39], with dimensions of 1″ × 12″ and a layup sequence of [45/0/−45/90]_2s_. These specimens were instrumented with four acoustic emission sensors as shown in Figure 2 and were quasi-statically loaded until failure. The results from two nominally identical specimens were examined in this paper. The specimens were subjected to quasi-static loading until failure, during which acoustic emission (AE) signals were recorded. To facilitate effective load transfer and prevent slippage, fiberglass tabs were adhesively bonded to both ends of the specimen. The load on the tensile specimen was ramped linearly until the specimen failed, and the duration of this loading was approximately one hour.

#### 2.1.2. AE Data Acquisition System

Data acquisition was performed utilizing the MICRO-II AE system, equipped with AE Win software (AEwin64™, 5.4.). A trigger threshold was set at 45 dB, complemented by an analog bandpass filter that included a 100 kHz high-pass filter and a 3 MHz low-pass filter to minimize mechanical noise interference. To detect AE signals effectively, four bonded PZT sensors, capable of acquiring signals having frequencies up to 3 MHz, were attached to the surface of the specimen.

A preamplifier with a gain of 40 dB was employed to amplify the signals received from the PZT sensors. The output voltage from the PZT was discretized at a sampling rate of 20 MSPS. The AE system had a maximum voltage recording limit of 10 V, which corresponds to a 100 dB level.

#### 2.1.3. Sensor Characteristics and Placement

A total of about 43,000 acoustic emission events were recorded by the four sensors bonded to test specimen 1 during the loading cycle. The sensors used were in-house PZT sensors bonded to the specimen at various locations, as illustrated in Figure 2. All four sensors of test specimen 2 recorded over 77,000 AE signals collectively. In the current research, signals from sensors 1 and 4 were excluded from further analysis to eliminate the possibility of noise interference generated by the grip, as they were located near the grip. In addition, they were used as guard sensors to confirm that the signals being analyzed are from damage modes, not mechanical noise. The results of the analysis of two datasets containing the AE signals collected from sensor 3 of test specimen 1 and test specimen 2 are presented in the paper.

### 2.2. Data Preprocessing

#### 2.2.1. Waveform Windowing and Selection

The number of preprocessed signals used as a dataset from specimen 1, sensor 3 was 14,546, and specimen 2, sensor 3 was 19,769. A sensor’s frequency response depends on its sensitivity, Lamb wave dispersion in the applied material (CFRP thermoplastic composites in this study), the distance between sensors, and structural attenuation. Hence, the same event can be recorded by sensors 2 and 3 and counted twice. Furthermore, the highest-frequency AE signals (>2000 kHz) are transient and difficult for a sensor to record if the damage source is not near it. They can be dispersed easily while traveling a longer distance. Therefore, sensor data may not capture the full range of signals. The two preferred datasets for this study included a dataset with a wide frequency range, allowing the researchers to investigate damage types. Results from sensor 2 of specimen 2 show that sensor 2 could not record any high-frequency signals (>2000 kHz) within the lowest-amplitude segment (45 dB–55 dB). These waveforms were recorded with a pre-trigger of 75 µs and a total duration of about 500 µs each. However, after examining a large number of these waveforms, a duration of 140 µs for each waveform, starting from 60 µs up to 200 µs of the recorded waveforms, was used in the detailed analysis to minimize noise inclusion in the signal. These 140 µs windows captured all the essential features of the waveforms originating from the various damage modes currently under study. High-frequency events corresponding to fiber-break signals had a relatively short duration compared to low-frequency delamination signals, which had a longer duration. The window size protected features from signals with diverse durations. Future work will include studying the sensitivity of the model to different window sizes.

#### 2.2.2. Amplitude-Based Segmentation Strategy

The first step of the mathematical model applies z-score normalization to the dataset. Each test dataset was segmented into five amplitude groups, as this approach represents a methodological trade-off where amplitude information is suppressed to enable the model to learn frequency and other temporal patterns that would otherwise be dominated by the amplitude variance. While amplitude correlates with the damage mode and severity, preliminary tests showed that processing all events together without amplitude segmentation resulted in amplitude-dominated features. This strategy preserves coarse amplitude information while suppressing fine-scale amplitude variations that interfere with frequency-based classification in the model. The segments were: 44–55 dB, 55–65 dB, 65–75 dB, 75–85 dB, 85–99 dB. The specific boundaries were chosen based on the AE acquisition system characteristics, with the lower bound representing the threshold and the upper bound the maximum digitization range. A 10 dB range was chosen to acquire gradual variations in classification (i.e., the number of classes) as amplitude increased. This partition allowed the mathematical model to identify the absence of the class with the highest frequency beyond 75 dB. It also helped identify the distinctive class with significantly high frequency content (around 800 to 1600 kHz) above 75 dB. However, since amplitude contains important information about damage severity, and the current model cannot provide information about the intensity of damage for each class within a specific amplitude segment, the authors intend to extend future research in that direction using alternative normalization schemes that preserve amplitude as an additional feature dimension while still enabling effective frequency-based classification.

This amplitude segmentation strategy represents a trade-off between preserving damage severity information and enabling the 1D-CNN to learn frequency/temporal patterns. 10 dB intervals offer a balanced sample distribution and clustering performance.

Preserved: Coarse amplitude information (which 10 dB range the event belongs to)

Suppressed: Fine amplitude variations within each 10 dB range (via z-score)

We acknowledge that this preprocessing discards potentially useful information. For example, a 52 dB and 54 dB signal (both in the 45–55 dB range) may represent different damage severities, but this distinction is lost after normalization. The 10 dB interval width was selected through preliminary analysis showing:

Finer intervals (<5dB): insufficient samples per group, unstable clustering;

Wider intervals (>15dB): excessive amplitude variance degraded 1D-CNN feature quality.

#### 2.2.3. *Z*-Score Normalization Methodology

This is the first step of the mathematical model. The preprocessed waveforms of each segment were fed to the mathematical model. The model initially applies *Z*-score standardization to the waveforms and then feeds them into the 1D-CNN model.

*Z*-score is defined as follows [41]:

(1) $$Z=\frac{x_{i}-\mu }{\sigma }$$

where *x*_i_ is the data point in the original waveform, µ and σ are the mean and standard deviation of the amplitudes.

### 2.3. Deep Learning Feature Extraction

**Figure 3:**
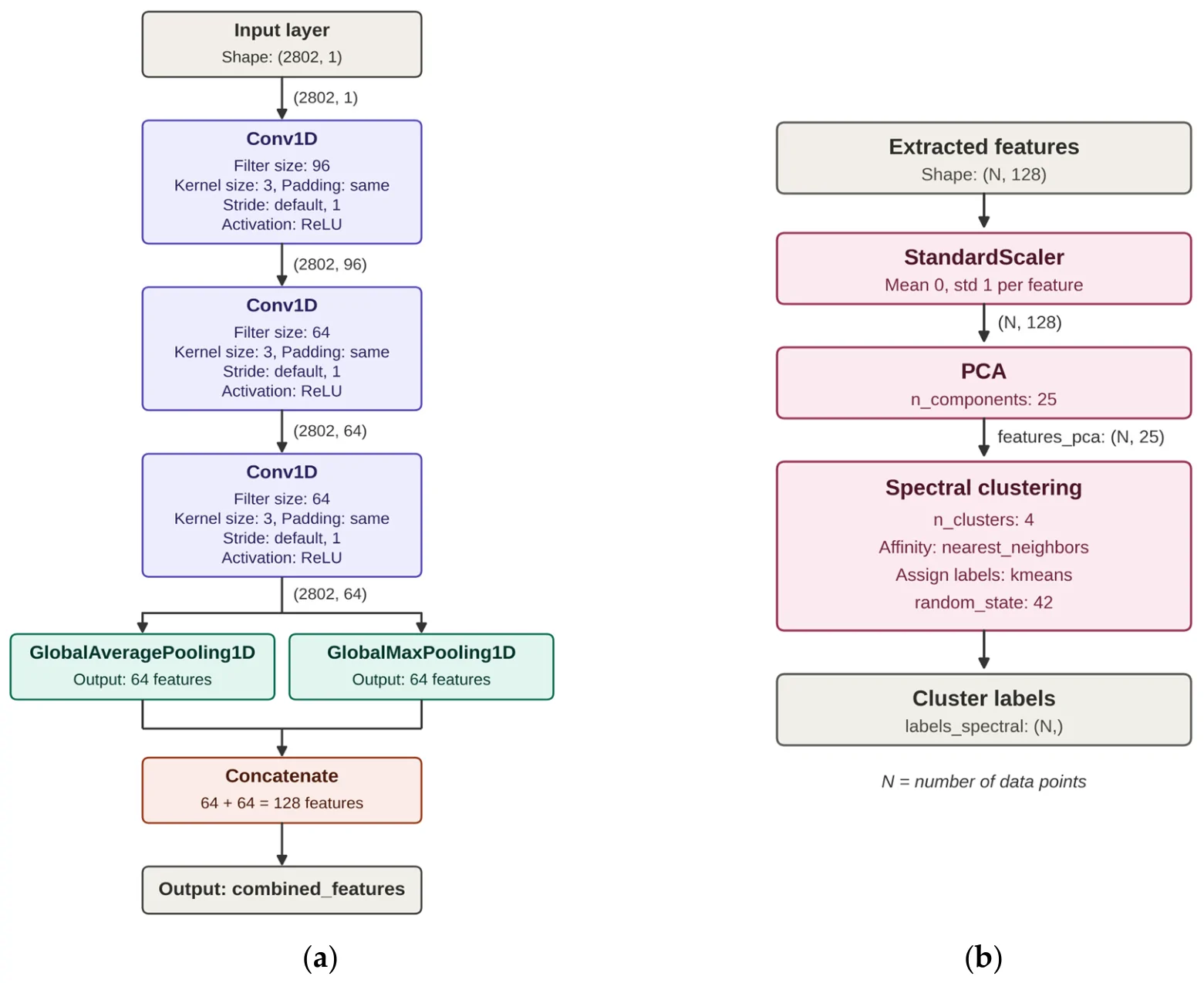
The deep learning-machine learning (DL-ML) framework, (**a**) feature extraction, (**b**) clustering (unsupervised learning).

Two-dimensional CNN [42,43,44,45] as well as 1D-CNN [46,47,48] have been used to classify the AE signals from damage in different types of composites, including thermoplastic CFRP composites. One-dimensional CNNs were initially used for speech recognition tasks with audio input data and began to gain popularity [49,50]. In the present approach, standardized waveforms were fed to the 1D-CNN model. Figure 3 illustrates the architecture of DL-ML frameworks.

To process such one-dimensional data, filters containing kernels are made to slide along the axis of the input vector [49,51]. For an input sequence x and a kernel size of three, the convolution operation is given as follows:

(2) *z_i_* = *w*_1_*x_i_*_−1_ + *w*_2_*x_i_* + *w*_3_*x_i_*_−2_

where $w={\left[w_{1},w_{1},w_{2}\right]}^{T}$ is the kernel. Filters were applied to extract significant characteristics of the sequence related to amplitudes, frequency, and other relevant features. The feature extraction framework consisted of three convolutional neural network (CNN) layers, with 96 and two 64 filters. Each filter had three kernels. The activation function ReLU can be written as follows:

(3) *z_c_* = *f*(*w_c_* ∗ *x* + *b_c_*)

where b_c_ is the bias term [49,51], and ReLU (x) = ReLU (0, x), although some researchers use leaky ReLU to consider the contribution of negative amplitudes; regular ReLU serves best for the current work among other activation functions. Instead of focusing on a single value, i.e., peak amplitude, the CNN model concentrated on the overall distribution of the amplitude and frequency. The feature maps from the three layers of the CNN were down sampled using global average pooling and global max pooling to retain the most essential segments of the information and reduce the spatial dimension [49,51]. After that, principal component analysis (PCA) of the features was performed to determine the most relevant features.

### 2.4. Dimensionality Reduction and Transformation of the Features

#### 2.4.1. Principal Component Analysis

Next, principal component analysis was performed on the features for each waveform to reduce the number of features and identify the most relevant features to classify the waveforms. The concept of principal component analysis was introduced by Pearson (1901) [52]. Later, Hotelling (1933) [53] developed the idea as an analysis tool for multiple variables in the dataset. Principal component analysis reduces dimensionality by eliminating interrelated variables and identifying the principal components that encapsulate the most variability among the signals considered. It is accomplished by transforming each of the waveforms into a new set of variables called principal components, which are linear combinations of the original variables. To reduce dimensionality, one must investigate the linear combination with maximum variance [54]. In the present problem, the waveform has ‘n’ data points, each of which has a value x_i_. The first step is to look for the linear function α_1_’x of elements of x having maximum variance, where α_1_ is a vector of n constants: α_11_, α_12_, …, α_n,_ and α_1_’x denotes the transpose of α_1_x so that

(4) $$\alpha_{1}’x=\alpha_{11}x_{1}+\alpha_{12}x_{2}+\ldots \ldots +\alpha_{1(n)}x_{n}=\sum_{j=1}^{p}\alpha_{1j}x_{j}$$

Here, α_1_’x is the first principal component (PC1). The maximum variance of each PC is uncorrelated with other linear functions or PCs. But it is expected that most variation in the variables will be accounted for by m number of PCs, such that m < n. In this research, results were reported for 25 PCs since 25 PCs include over 85% variance. However, the cumulative variance for principal components greater than 10 is almost the same. Effective clustering or partitioning of data depends on the features exhibiting most variance [15,54].

#### 2.4.2. Selection of Optimal Number of Components

Twenty-five principal components were carefully chosen to retain> 85% of cumulative explained variance while balancing computational efficiency. Empirical results (consistent cluster separation across two specimens) demonstrate that 25 PCs retain sufficient information for meaningful classification.

#### 2.4.3. Physical Interpretability of 1D-CNN Features and Principal Components

While deep learning frameworks are frequently critiqued as “black-box” classifiers, the feature space extracted by the 1D-CNN and transformed via PCA in this study directly maps to the physical parameters of acoustic wave propagation and stress wave mechanics.

1.0 Physical Meaning of 1D-CNN Convolutional Filters:

The initial 1D-CNN layer employs sliding convolutional kernels along the digitized voltage time series x(t). Rather than evaluating isolated scalar metrics (such as peak amplitude or threshold counts), the learned filter weights extract:

The local energy arrival rate (wavefront rise time τ_r_ = t_peak_ − t_onset_);

The dominant transient frequency spectrum (ω_d_);

The wave envelope attenuation/decay rate (α_d_).

2.0 Physical Meaning of Principal Components (PC1 and PC2 ):

Principal component analysis (PCA) maps the 128-dimensional pooled feature vector onto orthogonal axes capturing maximum variance (>85% cumulative variance). Physical inspection of the reconstructed waveforms across the PC coordinate space reveals clear physical proxies:

• Principal Component 1 (PC1) as a Spectral Energy Centroid Proxy: PC1 strongly governs partitioning along the frequency domain. Low or negative PC1 values (PC1 < −10) systematically correspond to high-frequency emissions (f > 1600 kHz), characterized by short acoustic wavelengths. High positive PC1 values (PC1 > 10) correspond to low-frequency, long-wavelength emissions (f < 250 kHz).
• Principal Component 2 (PC2) as a Temporal Duration Proxy: PC2 isolates the temporal decay profile and dispersion characteristics of the propagating Lamb wave modes. Lower PC2 values correspond to highly transient, impulsive signals with rapid exponential decay (characteristics of localized source events such as single-fiber fracture). Higher PC2 values capture extended, highly reverberating wave envelopes with long ring-down counts (characteristic of continuous matrix micro-cracking and interlaminar shear delamination propagating along the specimen length). This is further shown in the figures below when comparing waveforms from different dB ranges.

### 2.5. Unsupervised Classification

#### Spectral Clustering Algorithm

All waveforms, based on their N number of features or dimensions, were then classified, where N is the number of principal components in this approach.

Identifying and isolating the group of points that belong to a specific signal class is challenging in a multi-dimensional domain since the distributions can take complex non-circular shapes. Special techniques have been developed for the separation of these points in N-dimensional space. Of these, spectral clustering approaches were considered. The spectral clustering approach performs better than the k-means clustering approach. This clustering method utilizes graph partitioning and the eigenvectors of the similarity matrix [55,56] to cluster data. In this work, a ‘similarity graph’ is used to partition these points representing individual waveforms into groups that may represent different signal classes. The points in the graph are termed ‘nodes’, and these nodes are connected to other nodes through ‘edges.’ If the characteristics of two points representing two waveforms are similar, then they are connected by a highly weighted edge. If the two points are totally dissimilar, they may not be connected at all. A weight of ‘1’ for the edge connecting two points in the N-dimensional space would indicate that the two points are similar and hence close to each other. In this work, a KNN graph was used, where each node is connected to its *k* nearest neighbors. The model utilized the built-in KNN of the spectral clustering in the Python (Google colab: https://colab.research.google.com/) library. Hence, the default value was used by the algorithm. The spectral method employs graph Laplacian matrices for clustering [22,56].

After forming the similarity matrix A, the diagonal matrix D is defined to represent the connection degree of each node, and the Laplacian matrix L is then constructed. The number of zero eigenvalues corresponds to the number of connected nodes. The top *k* eigenvectors of L are extracted and arranged as columns, after which k-means clustering is applied to the rows of this matrix to identify clusters. Other clustering algorithms could also be used at this stage; however, k-means clustering was applied here.

Direct application of k-means clustering often fails to produce meaningful clusters because it assumes convex cluster shapes, which may not reflect the natural data structure [22,57,58].

In the current study, spectral clustering attempts to identify the natural groups utilizing 25 principal components as new features, unlike the regular unsupervised classification approach. Inspection of the fast Fourier transform (FFT) of the waveforms ultimately revealed that the classes vary mainly by frequency components.

### 2.6. Cluster Validation Metrics

Validation indices are tests to determine the optimum number of clusters. For the AE waveforms that are being analyzed in this paper, amplitudes of events ranged from 45 dB to 100 dB, and the dominant frequency of individual events ranged from 50 kHz to 3000 kHz, indicating the presence of widely different signal classes. Validation indices for numbers of classes ranging from 2 to 10 were examined to determine the optimal number of classes.

To evaluate the number of classes, the following cluster evaluation metrics were examined.

• The silhouette score,
• The Elbow plot,
• The Davies–Bouldin index (DBI),
• The Calinski–Harabasz index (CHI).

#### 2.6.1. Silhouette Coefficient

One of the most common metrics used for evaluating the performance of classification is the ‘Silhouette coefficient’. Clusters that contain highly similar waveforms, positioned very near each other in the N-dimensional space, are said to have high cohesion (a in Equation (5)) and will have a score of nearly 1. Cohesion refers to the average distance between a given data point and all other data points within the same cluster. Separation (b in Equation (5)) indicates the average distance between data points within a cluster and other data points in other clusters. [59]. The silhouette coefficient, s, is given as

(5) $$s=\frac{\left(a-b\right)}{\max\left(a,b\right)}$$

The overlapping boundary of the classes explains the lower silhouette coefficient. A silhouette score close to 1 refers to a well-separated class.

#### 2.6.2. Elbow Method (WCSS)

Another method commonly used to assess the performance of k-means clustering is the within-cluster sum of squares (WCSS), visualized via an elbow plot [60,61]. It is the sum of the squared distances between each point and its centroid. While WCSS is theoretically designed for centroid-based methods (k-means) and may not fully capture the quality of non-convex clusters identified by spectral clustering, we considered elbow plots to provide additional insight into cluster compactness. These should be interpreted alongside the more appropriate DBI and CHI metrics. Based on both the silhouette coefficient and DBI values, four clusters provided the best balance between separation and interpretability for amplitude ranges <75 dB that contained the maximum number of data points with wide variance.

#### 2.6.3. Davies–Bouldin Index (DBI)

However, the Silhouette score and Elbow plot are generally effective for k-means clustering [59,62] since k-means clustering is designed to find roughly ball-shaped clusters or classes. Davies et al. [63] presented a method to assess the quality of the clusters. It is called the Davies–Bouldin index (DBI). It measures the compactness of point distribution within each of these clusters as well as how far the individual clusters are separated from each other. This method is compatible with spectral clustering. The value for compact and well-separated clusters is expected to be close to 0. DBI is expressed as follows:

(6) $$\mathrm{DBI}=\frac{1}{k}\sum_{i=1}^{k}\max_{i\neq j}\frac{\left(S_{i}+S_{j}\right)}{M_{\mathrm{ij}}}$$

where S_i_ and S_j_ are intra-cluster dispersions, that is, the distance between each point and the centroid of cluster i, j, etc. M_ij_ is the inter-cluster separation, which is the distance between the centroids of cluster i and cluster j. k is the number of clusters in Equation (6).

#### 2.6.4. Calinski–Harabasz Index (CHI)

Calinski–Harabasz index (CHI) is another validation metric. It is expressed as follows:

(7) $$\mathrm{CH}\left(K\right)=\frac{B(K)(N-K)}{W(K)(K-1)}$$

where N is the number of samples, and K is the number of clusters. B(K) is a measure of the total squared distance between the center of each cluster and the center of all data, normalized by the number of points within each cluster. A large B(K) indicates that the individual clusters are well separated. Better separation has a higher CHI ratio [64,65].

#### 2.6.5. Interpretation Guidelines for Each Metric

In the initial attempt at finding the optimum number of clusters, when the entire set of waveforms was processed together, the optimal number was not clear, and the clusters were not well separated. In the modified process, AE waveforms were separated based on their amplitudes into the following five groups: 45–55 dB, 55–65 dB, 65–75 dB, 75–85 dB, 85–99 dB. The process was repeated individually for each of the five groups and found to be successful in forming clusters that maintained their characteristics across the different amplitude bands. Due to the physical relation between the damage mode and the frequency, in this work, the authors hypothesized that the number of clusters that developed along PC1 corresponds to the number of primary damage modes since PC1 correlates with the frequency of the data. From this attempt, four different classes of AE waveforms emerged. They were low-frequency events (around 50~250 kHz), mid-frequency events (around 250~900 kHz), mid-high frequency events (around 900~2000 kHz), and high-frequency events (above 2000 kHz). Low-frequency events typically correspond to delamination-type damage, mid-frequency events correspond to matrix crack-type damage, and fiber-break-type damage can be represented by mid-to-high and high-frequency events [10,12,13,14].

### 2.7. Waveform Similarity Analysis

#### 2.7.1. Cross-Correlation Methodology

Waveform correlation can provide a quantitative understanding of the identical type of damage at the exact location [11,66,67]. Signals having a cross-correlation coefficient greater than 0.9 can be considered to have the same source-to-sensor distance [68]. In this study, the fiber-break type of events (AE events with 700–1600 kHz and above 1600 kHz frequency components) that were obtained from the unsupervised clustering were considered for this test. Then, cross-correlation [69] was performed on them. The objective was to identify the maximum number of groups of fiber breaks that were correlated by a cross-correlation coefficient of at least 0.90, as the thermoplastic CFRP composites showed reluctance to form ‘multiplets’.

#### 2.7.2. Window Selection and Threshold Criteria

The duration of fiber-break-type events can be as small as 15 µs up to 30 µs. For cross-correlation calculation, information in the leading-edge portion of the signals holds the most importance. Furthermore, the noise can enhance the possibility of false positives at a coefficient of 0.9. Hence, in this study, we used a 15 µs window for each event, starting from the pre-trigger zone, to avoid correlation with noise.

The 15 μs window captures the leading edge and primary frequency content of fiber-break events while minimizing contamination from reflections. The 0.90 threshold balances sensitivity (detecting legitimate multiplets) with specificity (avoiding false positives from coincidentally similar waveforms).

## 3. Results and Discussion

### 3.1. Validation Metrics

**Figure 4:**
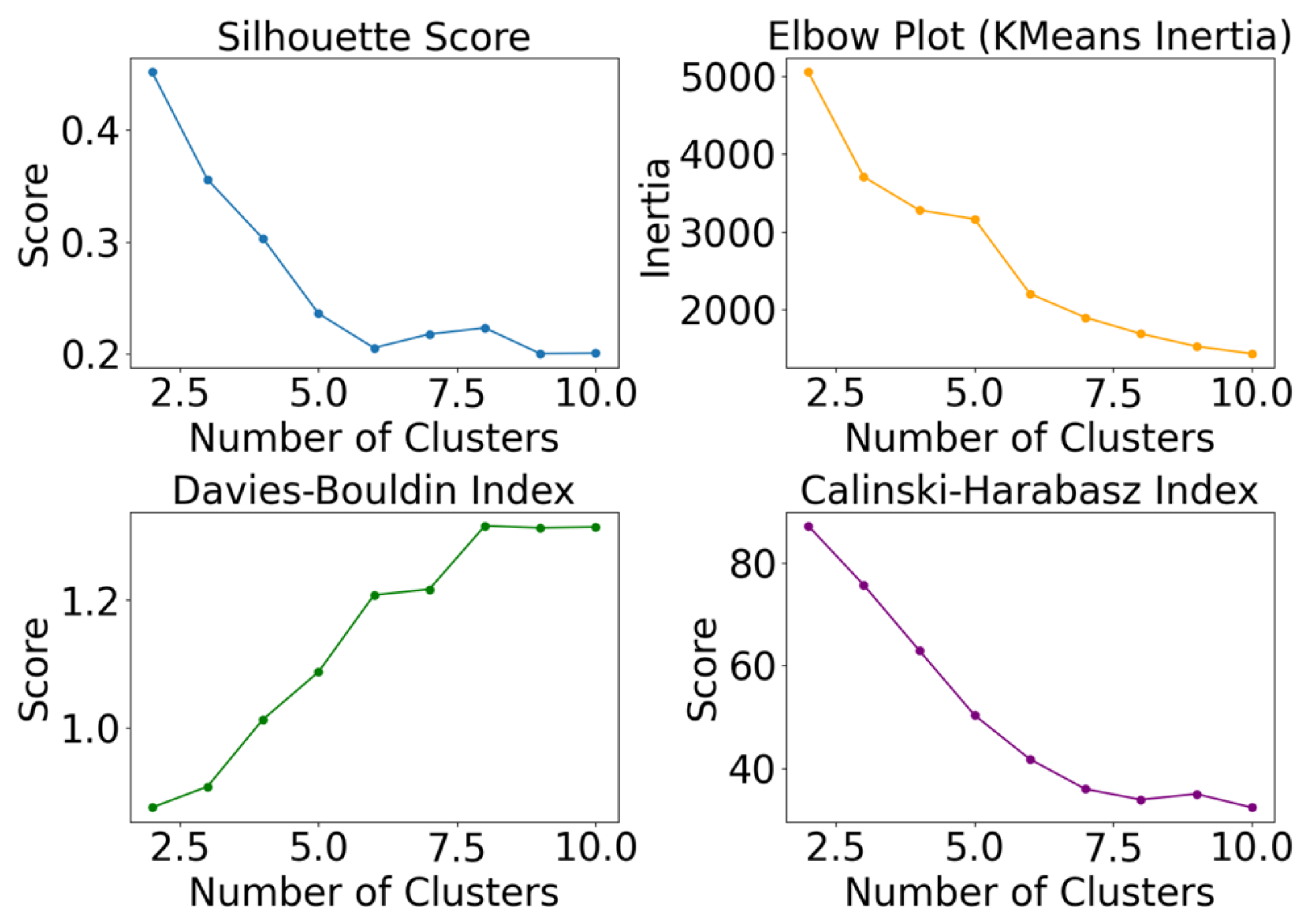
The scores of validity indices for different cluster numbers.

Figure 4 presents the plots that illustrate the variation in validity indices with respect to the number of classes of AE waveforms. Across the datasets analyzed in this study, the indices exhibit similar trends in each dataset. The validity measures show the highest separation when the data are partitioned into two classes, which likely corresponds to low- and high-frequency groups. However, the elbow plot suggests that data points within these two classes remain broadly distributed across the feature space, resulting in relatively high values. Increasing the number of classes enhances within-class compactness. Further examination of the class structure and the corresponding PC1 value ranges revealed that waveforms with amplitudes less than 75 dB are best separated into four classes, whereas waveforms with amplitudes greater than 75 dB could be separated into three classes. The fourth class of AE waveforms with amplitudes below 75 dB were very high-frequency events. This partitioning is supported by moderately low Davies–Bouldin (DB) index values and comparatively high Calinski–Harabasz index (CHI) values for cases in which an adequate number of waveforms within the amplitude range are available. A summary of the validation indices used in the selection of the number of clusters is illustrated below in Figure 4.

### 3.2. Cluster Visualization and Separation

**Figure 5:**
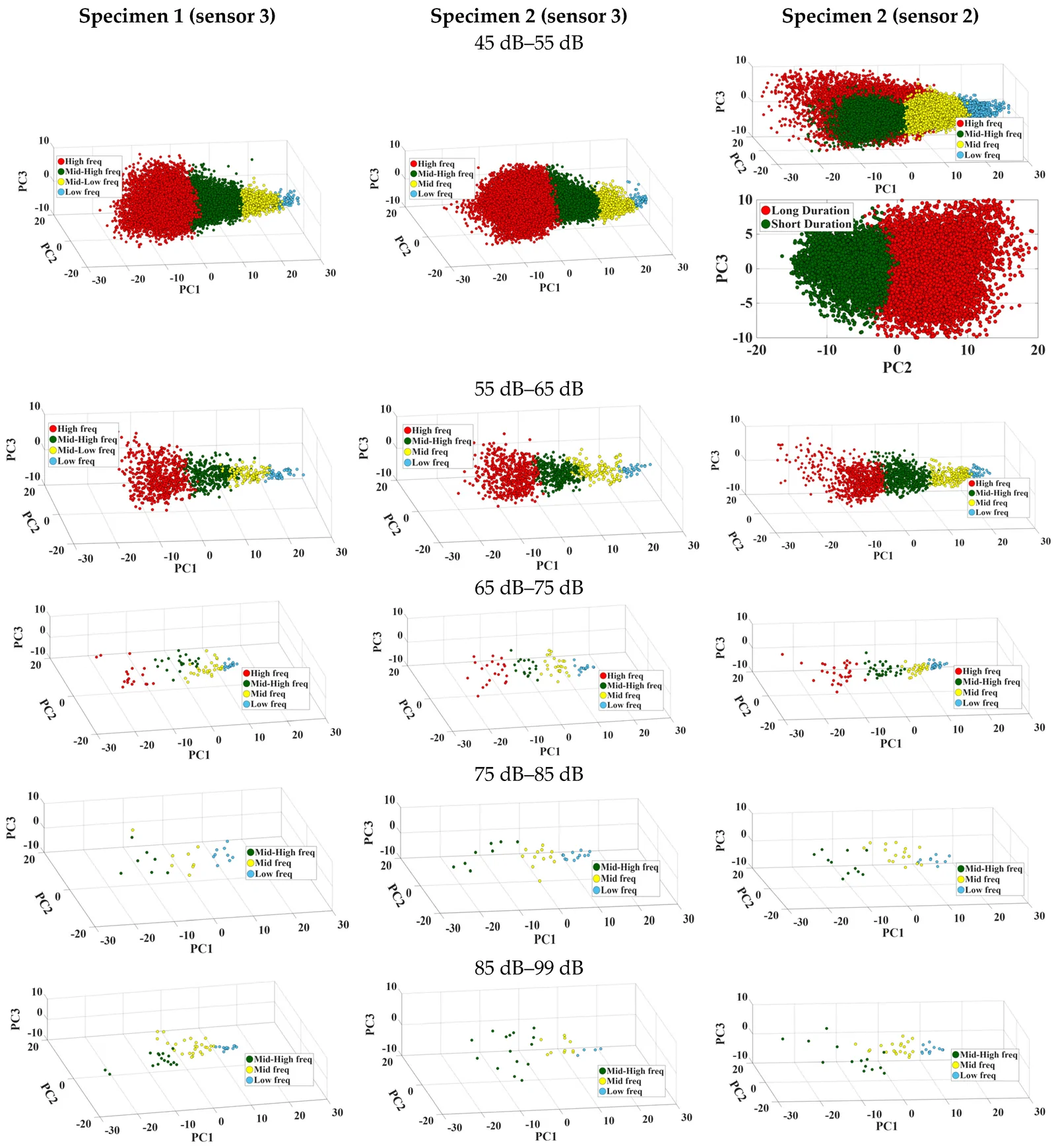
Cluster visualization plot.

Figure 5 presents the classification of AE waveforms into classes in the three-dimensional space represented by the first three principal components. As described previously in the data preprocessing section, AE waveforms were first separated into five distinct amplitude ranges as shown and then classified based on their principal components using unsupervised classification. The efficiency of clustering improved when the events were segmented into separate amplitude ranges. These clusters appeared to be compact and well separated along the first principal component axis. Detailed examination of members of each group revealed that frequency and duration of AE signals are the main attributes distinguishing the different groups. The first principal component values appear to be inversely correlated with the dominant frequency of AE waveforms. The second principal component values appear to be correlated with the duration of AE waveforms. Similar 3D plots with respect to higher order PC did not result in any separate class for the current datasets.

The figure also compares the sensitivity of two sensors on a specimen. The data from sensor 2 of specimen 2 failed to obtain a distinct high-frequency cluster. Rather, the high-frequency cluster overlapped with the mid-high-frequency cluster along PC1 within the 45 dB–55 dB segments, indicating similarity in the frequency content of both classes. This may be due to limited sensor sensitivity or dispersion of the high-frequency signals.

Based on frequency characteristics and correlations with published literature on composite damage [12,13,14], we propose the following interpretations:

• Low frequency (50–250 kHz): likely delamination-type damage;
• Mid frequency (250–900 kHz): likely matrix cracking;
• Mid-high frequency (900–1600 kHz): potentially clustered fiber breaks;
• High frequency (>1600 kHz): likely individual fiber breaks.

These interpretations remain hypotheses requiring validation through:

(1) Synchronized in situ imaging (micro-CT, high-speed camera);

(2) Acoustic source location with post-test microscopy correlation.

The consistency of these frequency-based clusters across two independent specimens provides preliminary support for these assignments.

**Figure 6:**
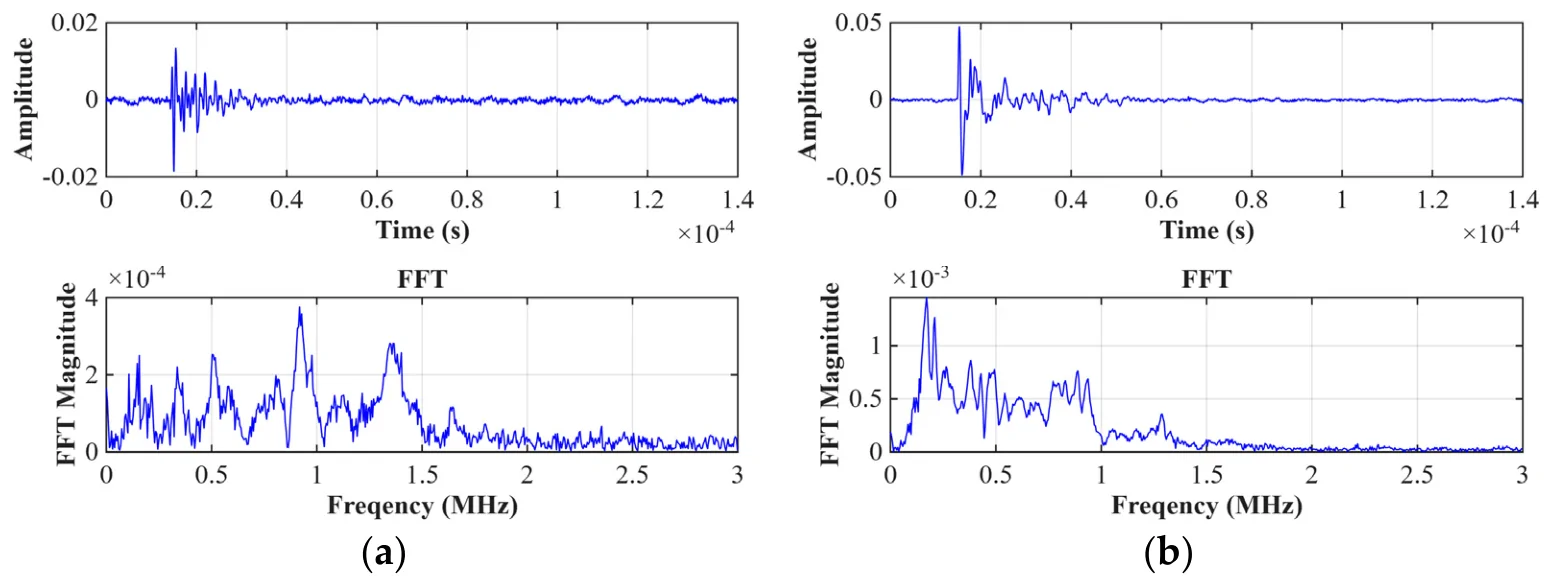
Events from 45–55 dB amplitude segment of Specimen 2, Sensor 2: (**a**) PC1 = −23.92, PC2 = 13.997; (**b**) PC1 = −20.62, PC2 = −12.26.

**Figure 7:**
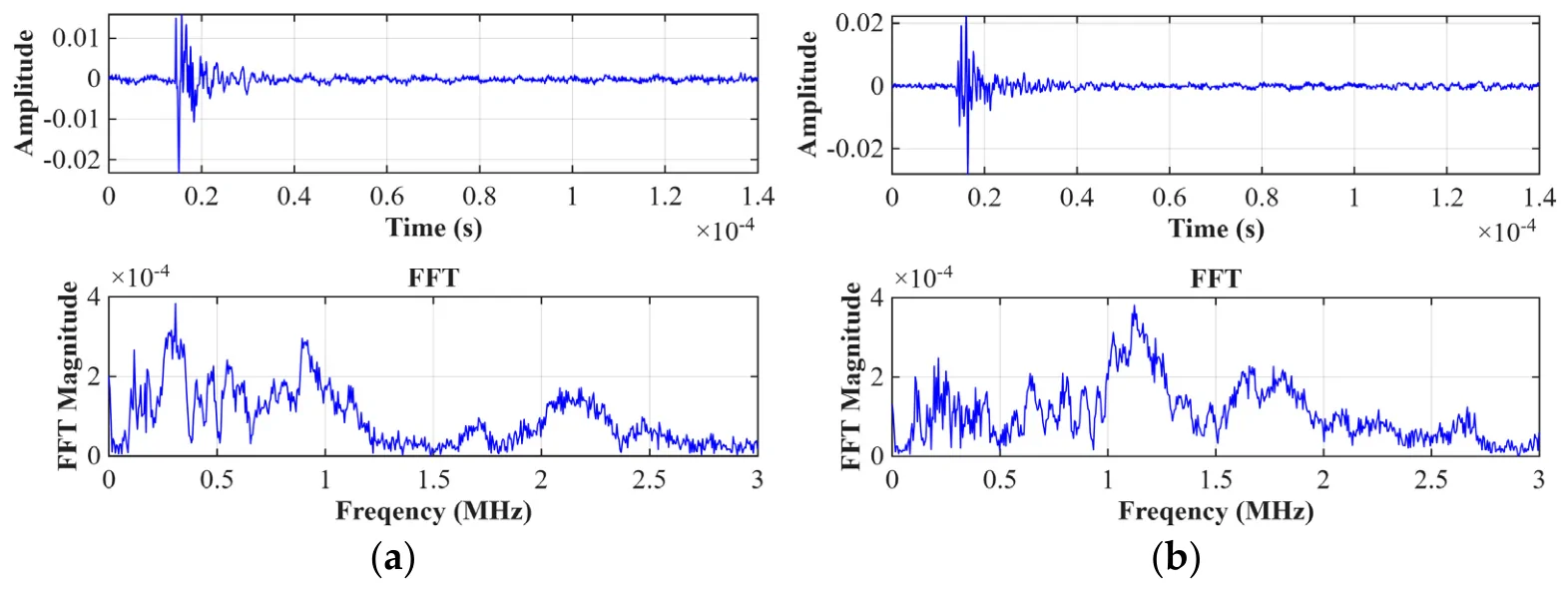
Events from 45–55 dB amplitude segment: (**a**) test specimen 1, PC1 = −10.0579; (**b**) test specimen 2, PC1 = −10.0526.

The two waveforms in Figure 6 belong to the same sensor group (Sensor 2 of Specimen 2). With similar PC1 values and contrasting PC2 values, the two waveforms indicate a correlation between PC2 and duration. The two waveforms are close in frequency but differ in duration. The example evidently shows that sensor 2 for specimen 2 could not record the unique high-frequency AE signals with the lowest amplitudes. The contributing reason may incorporate the dispersion of the signals and attenuation in the PAEK matrix materials due to the source-to-sensor distance. Figure 7 demonstrates the comparative views of the resemblance between the FFT of the waveforms with nearly identical PC1 from the two specimens, both belonging to the high-frequency class with low amplitude.

**Figure 8:**
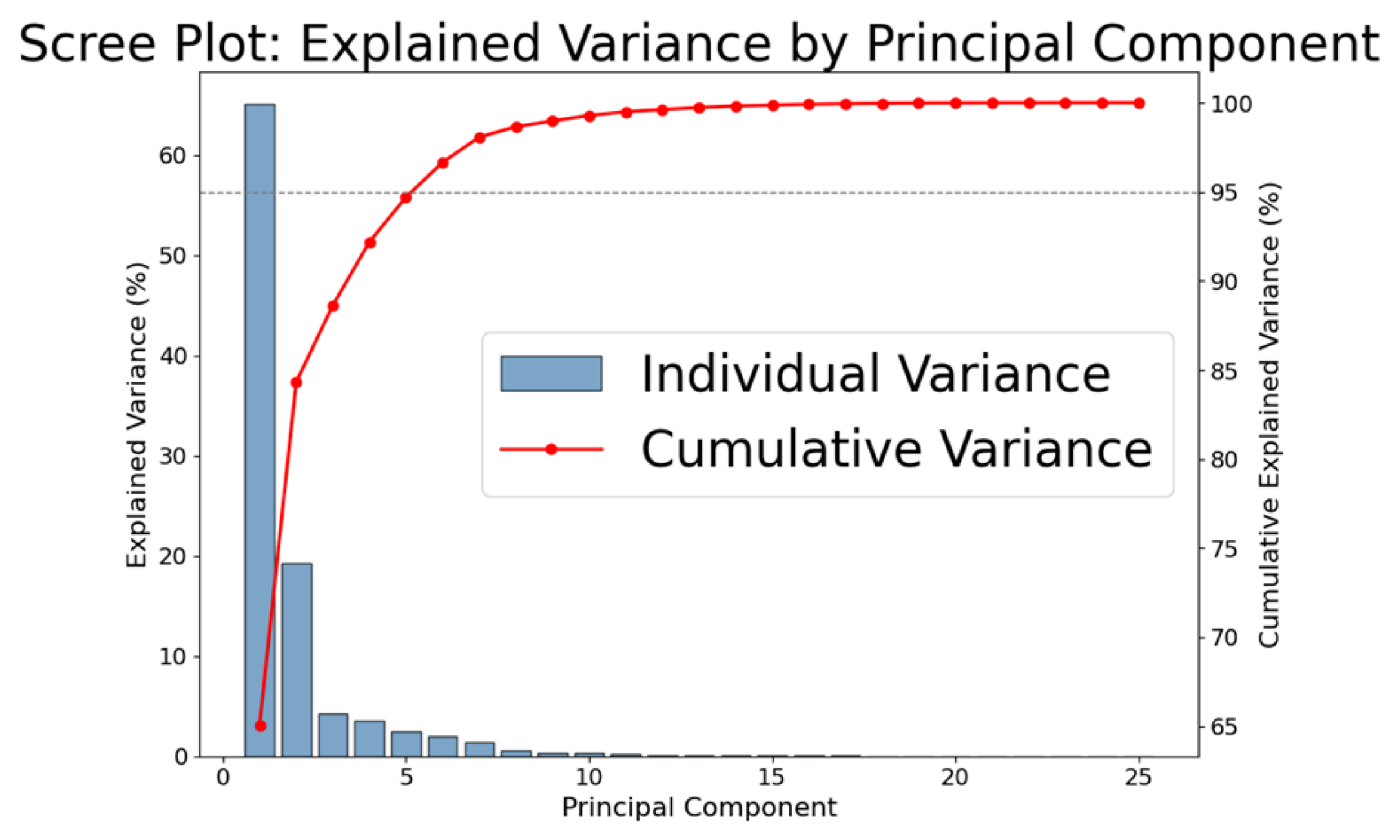
Scree plot.

Figure 8 presents the scree plot showing the variance in each principal component, with comparatively higher variance in the first principal component, as shown in the plots in Figure 5.

### 3.3. Frequency-Based Characterization of Clusters

#### Tripartite Mapping Between Model Features, Acoustic Responses, and Failure Mechanisms

To establish a deterministic relationship between the unsupervised model clusters and physical damage progression, a tripartite mapping scheme was established linking:

• Model feature signatures (PC1, PC2);
• AE signal attributes (frequency, Lamb wave mode);
• Microstructural damage mechanics.

Table 1 details the explicit mapping established for the [45/0/−45/90]_2_*_s_* PAEK thermoplastic carbon composite system.

**Figure 9:**
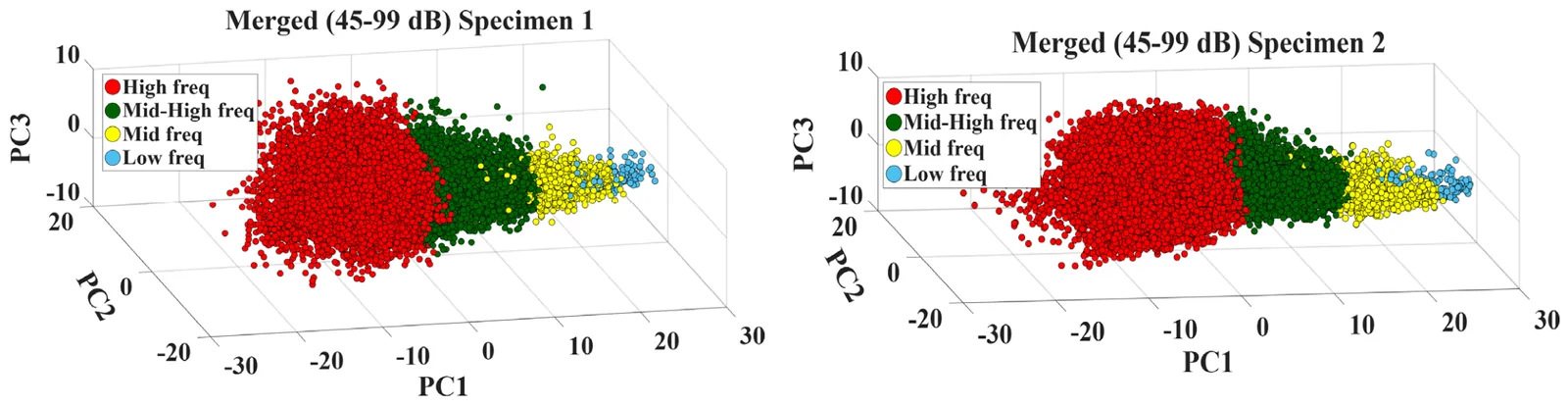
Cluster visualization plot for 45–99 dB for specimen 1 (**left**), specimen 2 (**right**).

Figure 9 represents a visualization of all classes for dataset merging across all amplitudes (45 dB–99 dB) in the three principal components’ domain for both specimens. The collection of events in each class in the merged plot, as well as among those in individual amplitude bands, was remarkably similar. In addition, the results from two different specimens were also similar. The characteristics of events within each class of AE events were examined. Similarity in terms of AE parameters was maintained for events within individual classes across the two specimens.

**Figure 10:**
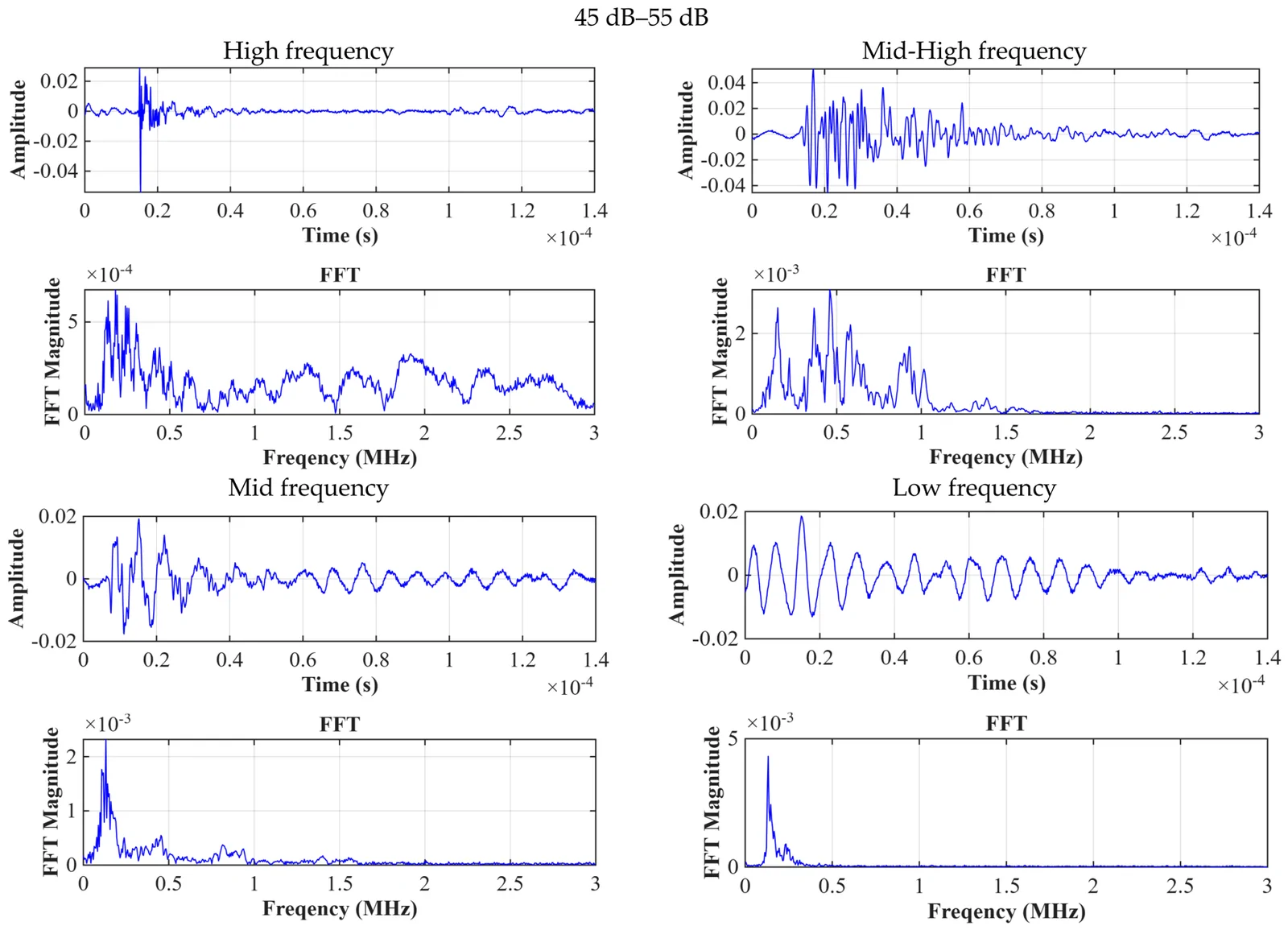

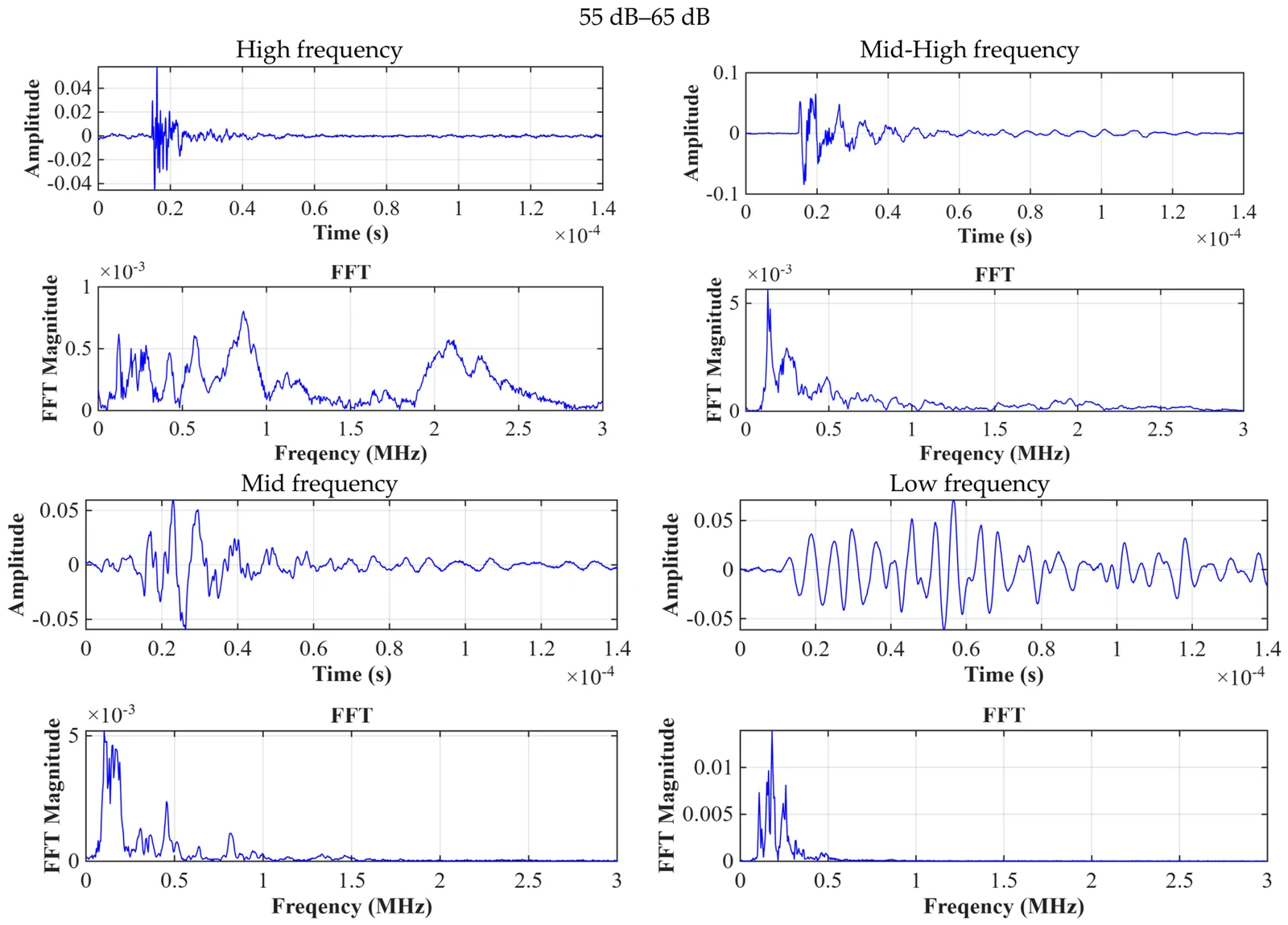

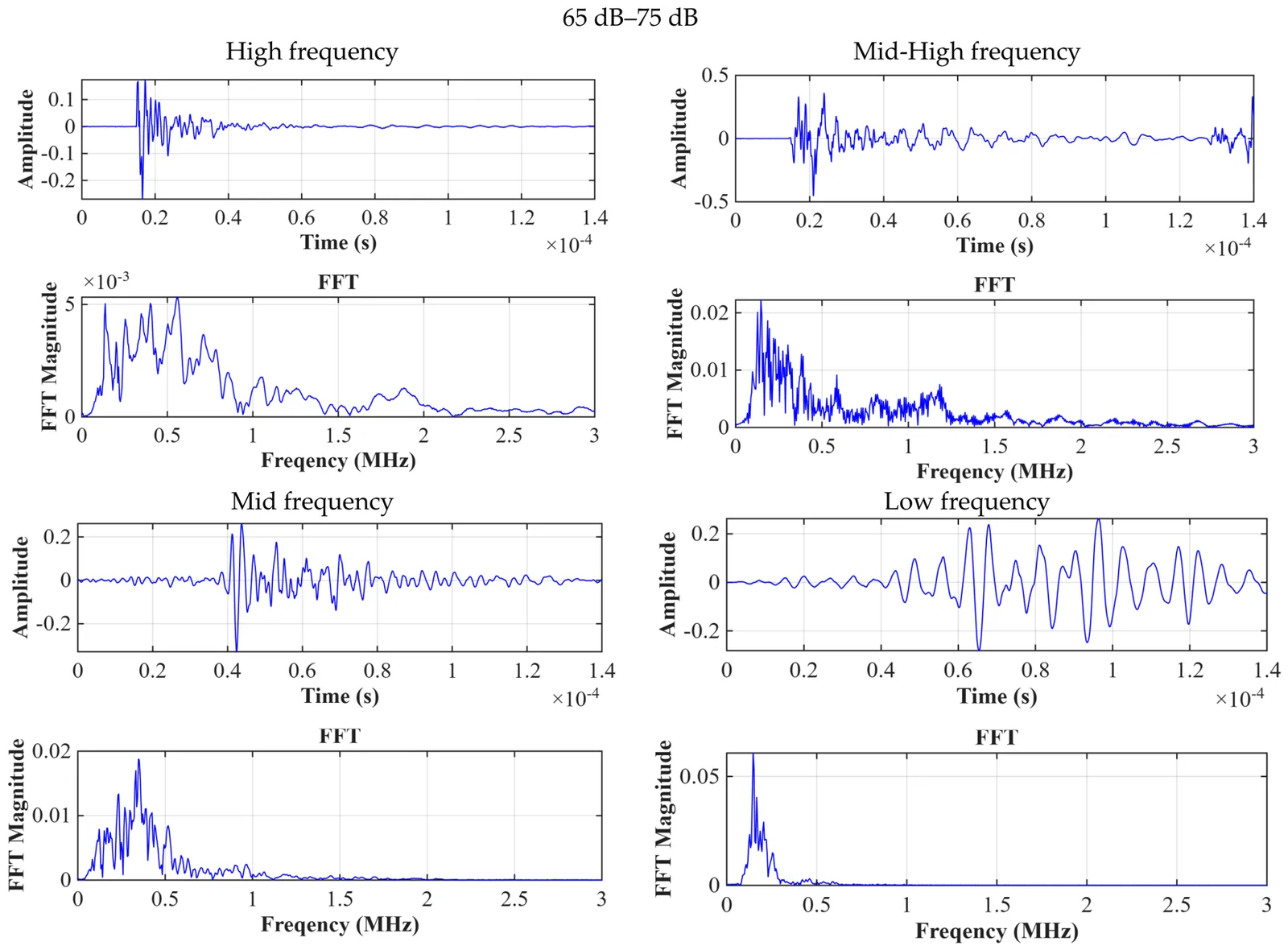

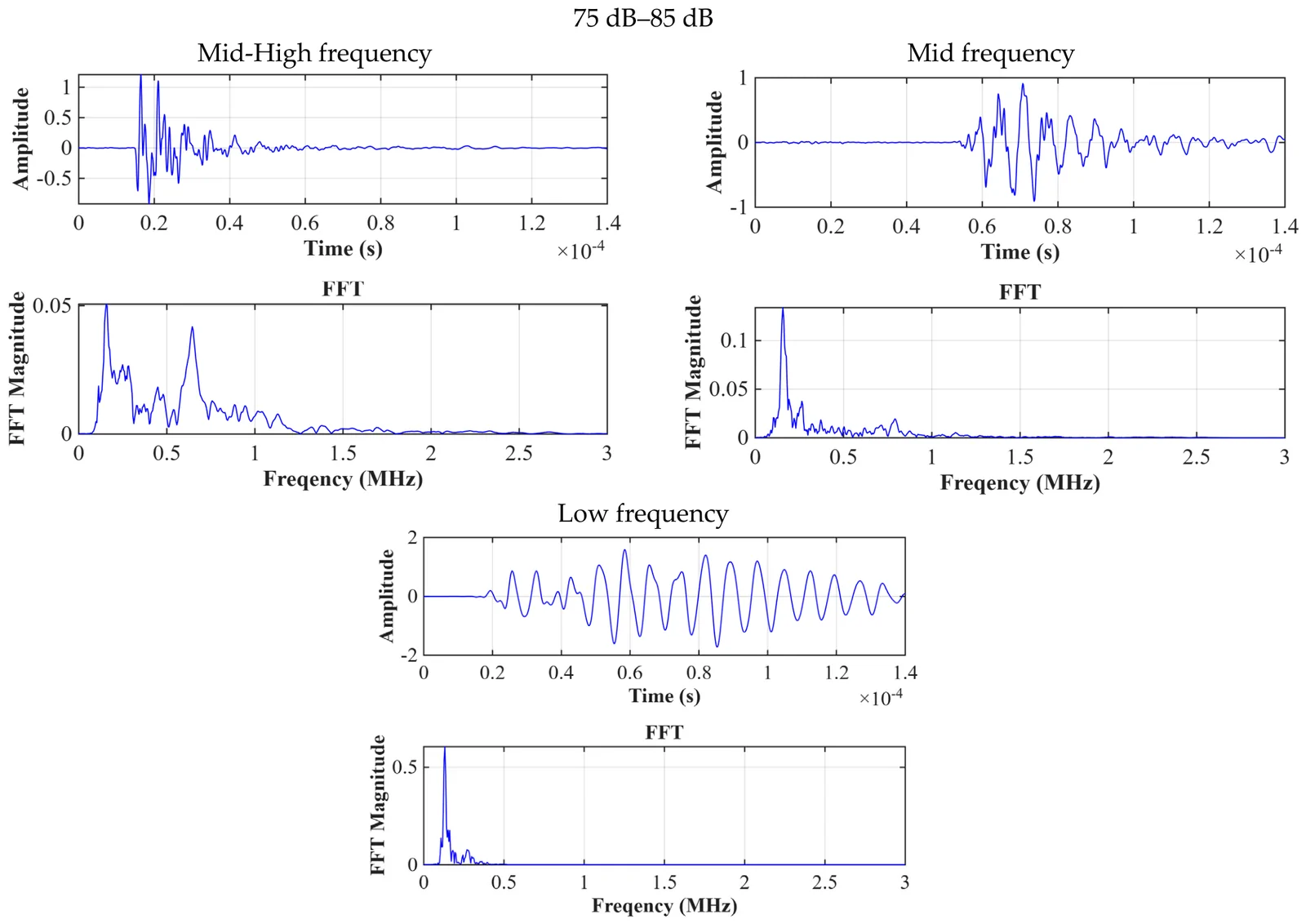

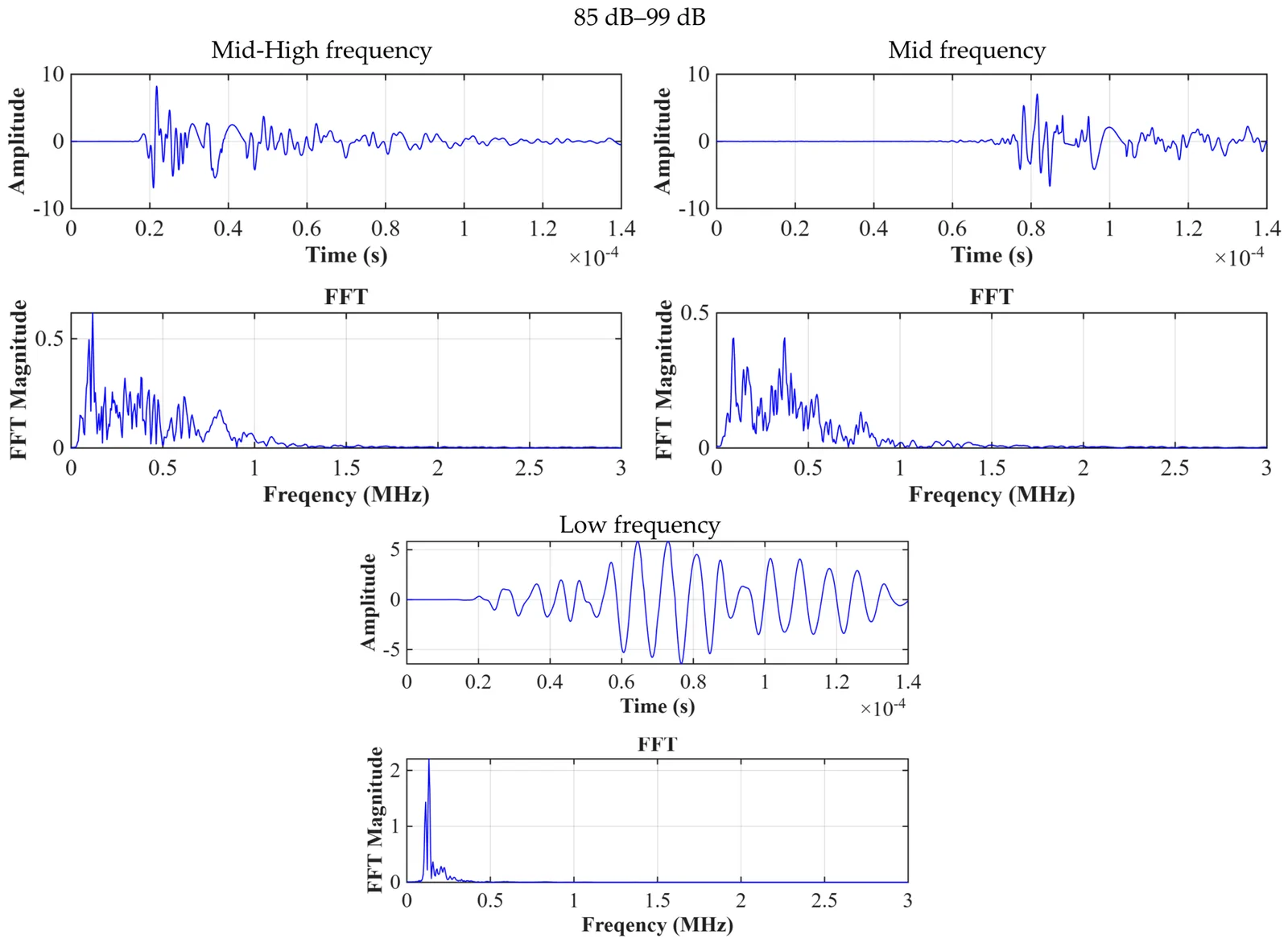
Class representative waveforms.

Figure 10 exhibits representative waveforms corresponding to different classes. The classes have been found to be distinct in terms of frequency components and duration. This uniformity in the classified groups adds credence to the approach employed.

**Figure 11:**
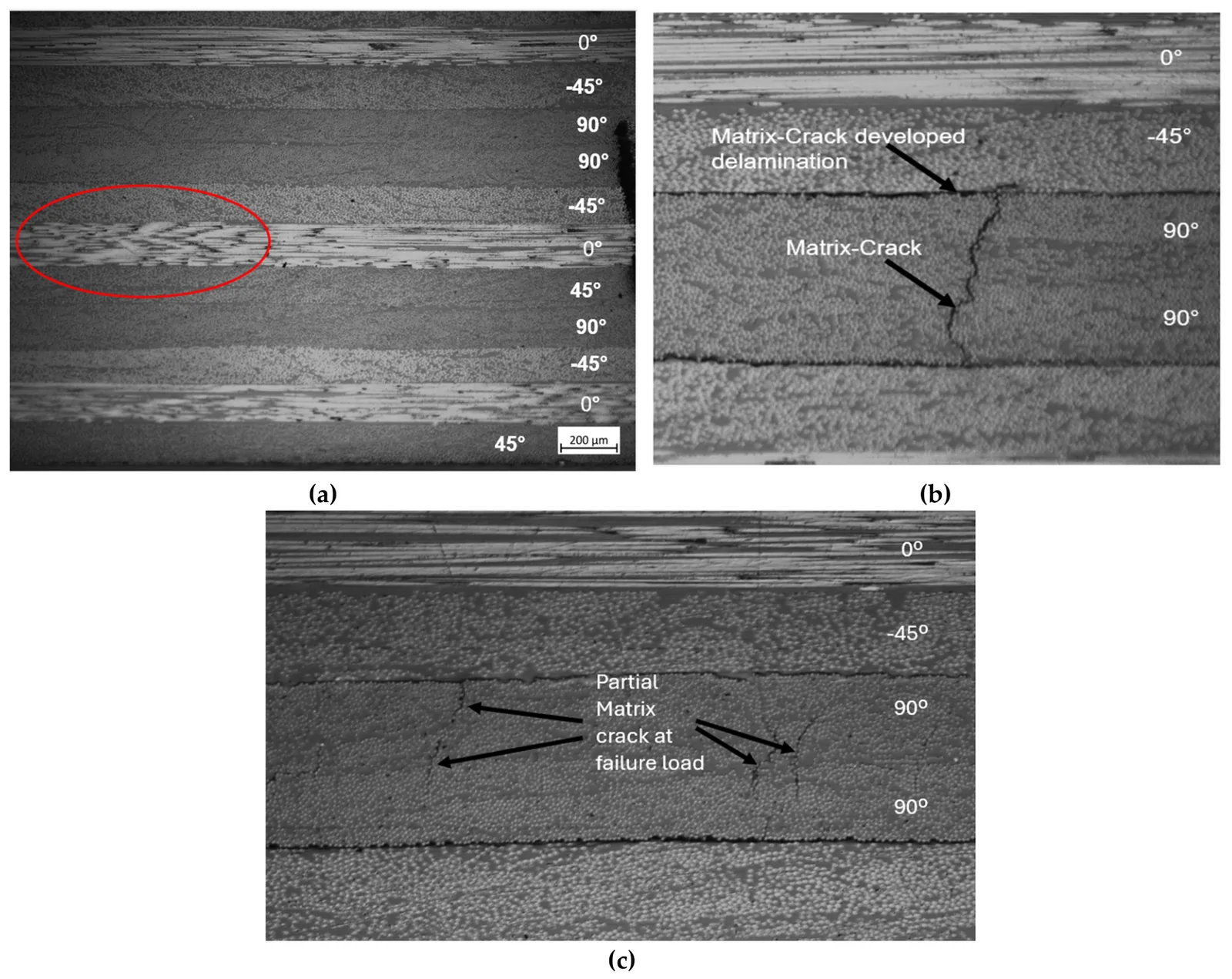
(**a**) Fiber waviness in the 0° layer in carbon/PAEK thermoplastic composites, (**b**) matrix crack-developed delamination, (**c**) partial matrix crack.

The mid-high frequency class (900–1600 kHz, 75 dB up to 99 dB amplitude), observed in PAEK-type thermoplastic CFRP composite specimens, has not been widely reported in thermoset CFRP composite literature. We hypothesize this may be associated with fiber waviness in 0° plies, which is more prevalent in thermoplastic composites due to manufacturing processes. A break in a group of wavy fibers can emit this moderately high-frequency, high-amplitude waveform. Optical micrographs confirm the presence of in-plane fiber waviness as shown in Figure 11a below. However, we have not established a causal link between waviness and these specific AE signals. This proposed mechanism is speculative and based on:

• Absence of similar signals in thermoset CFRP composite studies;
• Theoretical potential for localized multi-fiber breaks in wavy regions;
• Intermediate frequency range between matrix damage and single fiber breaks.

### 3.4. Microscopic Damage Observations

The following optical micrographs from Figure 11 show the in-plane fiber waviness in the 0° layer, as well as matrix cracks and matrix-crack-developed delamination. To rigorously validate the signal assignments from the unsupervised 1D-CNN spectral clustering model, post-mortem optical microscopy and wave displacement physics were synthesized.

Wave Mechanics of Individual Carbon Fiber Fracture (Class 1, >1600 kHz):

An isolated carbon-fiber break represents a microscopic point-source impulse. The dynamic micro-displacement (u(x, t)) at the fracture site is modeled as an elementary double-force dipole moment tensor M_ij_ [70]. Because the cross-sectional area of a single T700 fiber is minuscule (∼0.44 mm^2^) (TORAY, “T700S Carbon Fiber Data Sheet,” 2024), the source rise time is extremely short (*τ_s_ <* 0.1 μs). According to spectral wave theory, the frequency content of the radiated elastic wave is inversely proportional to the source rise time (*f_max_ ∝* 1/*τ_s_*). Consequently, single fiber breaks physically generate ultra-high frequency acoustic pulses (1.6–3.0 MHz) characterized by low amplitude due to the small individual strain energy release (∼nano-Joules).

Wave Mechanics of Wavy Fiber Bundle Fracture (Class 2, 900−1600 kHz):

Microscopic inspection (Figure 11a) reveals localized in-plane 0-degree ply waviness. The induced shear stress causes matrix micro-yielding and local loss of lateral support. When the critical strain is reached, multiple adjacent fibers in the wavy bundle break nearly simultaneously (cooperative failure). The involved source volume is substantially larger than a single fiber break, releasing significantly higher strain energy (producing amplitudes > 75 dB). However, because the rupture process propagates across the bundle width over a slightly longer duration, the peak frequency drops into the intermediate band (900–1600 kHz).

Validation of Matrix Cracking and Delamination (Classes 3 and 4, <900 kHz):

• Transverse Matrix Cracking (Figure 11c): Cracks propagating parallel to fibers in 90-degree plies generate primarily in-plane motion, exciting the symmetric extensional mode (S_0_) with dominant frequencies between 250–900 kHz.
• Interlaminar Delamination (Figure 11b): Delamination growth involves crack propagation along the ply interface, inducing large out-of-plane surface displacements. This directly excites the asymmetric flexural Lamb wave mode (A_0_). Due to the large flexural mass displacement and low stiffness in the thickness direction, A_0_ modes inherently propagate at lower phase velocities and lower frequencies (50–250 kHz).

### 3.5. Damage Evolution During Loading

#### Mechanical Response Analysis and Fracture Physics of PAEK vs. Thermoset Matrices

**Figure 12:**
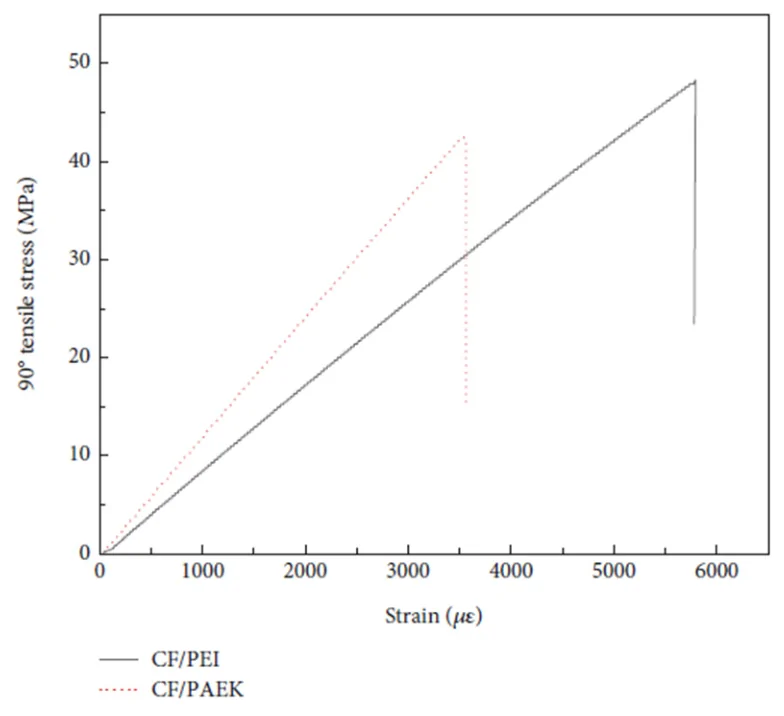
Typical 90° tensile stress–strain curves of CF/PEI and CF/PAEK composites [71].

**Figure 13:**
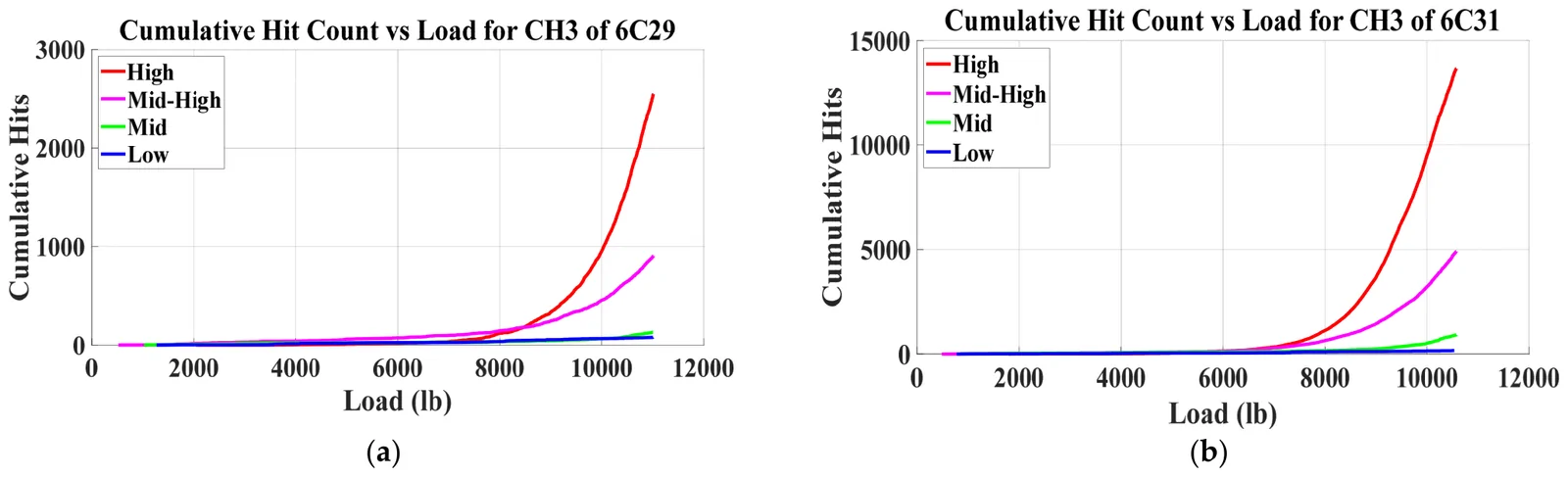
Cumulative number of events for different load levels for (**a**) specimen 1, (**b**) specimen 2.

Figure 12 shows the typical 90° tensile stress–strain curves of CF/PAEK composites [71]. The progressive accumulation of classified AE events across the tensile loading history in Figure 13 exhibits a correspondence with the multi-stage mechanical deformation behavior of quasi-isotropic PAEK thermoplastic composite laminates.

1. Stage I: Elastic Regime (0–30% Ultimate Tensile Load):
  At this stage of loading, the laminate exhibits linear elastic behavior. AE activity is virtually non-existent, confirming the absence of parasitic mechanical noise due to the effective guard-sensor filtering and 45 dB acquisition threshold.
2. Stage II: Matrix Yielding and Micro-Cracking (30–70% Ultimate Tensile Load):
  The onset of Class 3 events (250–900 kHz) occurs around 30–35% UTS, corresponding to transverse micro-cracking in the off-axis 90 degrees and ±45 degrees plies.
  Physical Delineation from Thermoset Composites: Notably, the total cumulative count of matrix-dominated events (Classes 3 and 4) in PAEK composites is lower by nearly an order of magnitude compared to standard carbon/epoxy (thermoset) systems reported in the literature [14,67]. This behavior is directly governed by the physical properties of the polyaryletherketone (PAEK) matrix. Wang et al. discussed this behavior [71] and found that better plastic deformation capacity can reduce stress concentration, forming more microcracks and thus absorbing more energy.
  • PAEK is a semicrystalline thermoplastic characterized by high fracture toughness. Wang et al. reported G_IC_ ~1786 MPa and G_IIC_ ~2584 MPa [71].
  • Under tensile shear, PAEK undergoes localized plastic shear yielding and molecular chain sliding prior to crack nucleation. The interlaminar shear strength of CF/PAEK is 90 ± 0.5 MPa [71].
  • This plastic deformation dissipates mechanical energy without generating high-amplitude elastic transient waves, resulting in fewer detectable low-frequency acoustic events.
3. Stage III: Fiber Alignment, Localized Waviness Failure, and Final Rupture (70–100% Ultimate Tensile Load):
  • Class 2 Evolution (900–1600 kHz): Class 2 events initiate steadily beyond 50% UTS and plateau near 80% UTS. As shown in the optical micrograph (Figure 11a), thermoforming and consolidation of thermoplastic tape laminates frequently induce in-plane fiber waviness in the nominal 0-degree piles. Under axial tension, wavy fibers experience severe localized matrix shear and secondary bending stresses, causing localized group-fiber fracture at lower global strain thresholds than straight fibers.
  • Class 1 Explosive Surge (>1600 kHz): Beyond 80% UTS, Class 1 events surge exponentially. In this regime, transverse matrix cracking saturates, and load is transferred directly to the load-bearing 0-degree carbon fibers. The fracture of individual T700 carbon fibers (d ≈ 7 μm) (TORAY, “T700S Carbon Fiber Data Sheet,” 2024) releases localized strain energy within nanoseconds, exciting high-frequency S0 Lamb wave modes (>1.6 MHz).

Because the ductile PAEK matrix provides high interfacial shear strength (IFSS), it delays macroscopic matrix splitting, forcing carbon fibers to carry load up to their true statistical strength distribution, leading to the observed surge in high-frequency fiber-break signals immediately preceding catastrophic failure.

### 3.6. Multiplet Analysis

**Figure 14:**
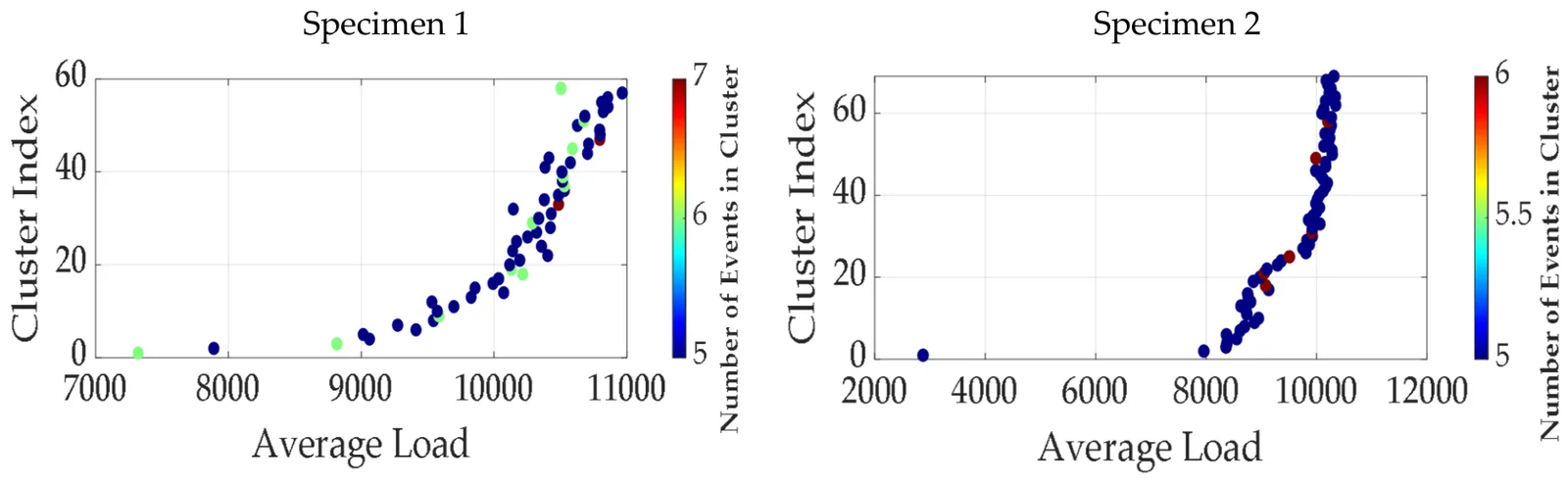
Cross-correlation among the fiber-break-type AE signals.

Several researchers have noted that near-simultaneous fracture of four or more adjacent fibers indicates the onset of unstable damage under tensile load in thermoset CFRP composite [67]. Whitlow et al. first noted that the onset of such instability is indicated by the appearance of AE events with nearly identical waveforms, and he plotted the clusters of near-identical waveforms as a function of tensile load. The number as well as the size of clusters of such near-identical AE events increased exponentially very close to the ultimate load in thermoset CFRP composite tensile specimens. As Figure 14 shows, PAEK thermoplastic CFRP composite generated fewer such clusters, and cluster sizes were also smaller. The higher ductility of the matrix material may be responsible for preventing fiber damage spreading in the lateral direction.

## 4. Summary and Conclusions

The evolution of damage in [45/0/−45/90]_2s_ PAEK thermoplastic CFRP composite materials under quasi-static tensile load is examined using the acoustic emission technique with the objective of identifying the different signal class and their relative participation as the load gradually increases. The features necessary for classifying the AE source mechanisms were extracted directly from AE waveforms, instead of relying on traditional AE features such as AE amplitude, duration, etc. The first part of the model extracted features from AE waveform data using a 1D convolutional neural network. Such feature extraction from data across multiple tests suggested that these features were unaffected by specimen-to-specimen differences.

The relatively large number of features generated by the 1D convolutional neural network was reduced further using principal component analysis. The second part of this work used spectral clustering to classify the acoustic emission events, based on principal components. The classification divided the projection of AE waveforms in the principal component space into four classes. The classes mainly exhibited separation along the first principal component direction.

The first principal component was found to be inversely related to the dominant frequency of AE events, suggesting that the underlying signal class may be separated in terms of the dominant frequencies.

As with any study, the approach presented in the paper carries certain limitations. The model performed better with z-score standardization, which loses amplitude (damage intensity) information. To retain this information, the data were partitioned into several amplitude groups before applying z-score standardization.

Previous studies [14,72] on thermoset CFRP composites subjected to tensile loading suggested that three types of AE signals related to three distinct failure modes, namely delamination, matrix cracks, and fiber breaks, were recognizable. These AE signals were associated with low frequency (100 kHz~250 kHz), medium frequency (250 kHz~650 kHz), and high frequency (>700 kHz) regions, respectively. An important observation regarding critical damage in thermoset CFRP composites based on microscopy and CT scans [73,74] is the formation of clusters of adjacent fiber breaks, termed ‘multiplets’, close to the final failure. The appearance of near-identical AE waveforms obtained by a sensor was thought to be an indication of the formation of such multiplets. Multiplets of order 10 or more were seen in thermoset CFRP composite. However, in PAEK thermoplastic CFRP composite tensile coupons, higher order ‘multiplets’ were not present. Testing the thermoset CFRP composite with similar features is required to support the hypothesis that formation of such ‘multiplets’ appears to be suppressed by the PAEK matrix material.

In the current study of PAEK thermoplastic CFRP composite quasi-isotropic tensile coupons, four classes of AE waveforms were recognizable based on frequency content. The frequency bands seen in the classification were low (50 kHz~250 kHz), medium (250 kHz~800 kHz), medium-high (800 kHz~1600 kHz), and high (1600 kHz~3000 kHz) frequency bands. There were relatively fewer events in the low- and medium-frequency bands seen in PAEK thermoplastic CFRP composite compared to thermoset CFRP composite. This may be related to the lower level of AE generation in the soft PAEK matrix material. Due to the Lamb-wave dispersion, source-to-sensor distance, and structural attenuation, the actual number of matrix crack and delamination-generated signals may be even less. Most of the high-frequency events were smaller amplitude signals. The high-frequency signals seen in the PAEK thermoplastic CFRP composite had higher frequency and were more numerous compared to those seen in the thermoset CFRP composite. While considering the above relative comparison between the two composite types, several factors that constrain generalizability should be kept in mind, such as the test setup and the lack of classification results for the thermoset CFRP composite using the same model. This highlights the future scope of the work: testing both composite types to confirm the hypothesis under the same conditions.

However, it may be noted that the PAEK matrix material is able to extract about 11% higher strength from the Toray fiber T700 compared to the epoxy matrix [75], indicating that carbon fibers in PAEK composites fail at higher stress levels, which in turn results in higher-frequency AE waveforms and eventually explains the unusually high frequency of the fiber-break-type events received in the PAEK thermoplastic CFRP composite.

Another limitation of the study is that the source mechanism of the medium-high frequency AE signals is not clearly established. Some of these signals have very high amplitudes. The fiber waviness seen in carbon-reinforced PAEK composites may be a likely cause of the medium-high frequency signals. Groups of fibers within this wavy region of the 0-degree lamina breaking together may be the source giving rise to such signals. Additional studies are needed to confirm these observations and better understand the failure modes in PAEK thermoplastic CFRP composite material.

Several future research directions could significantly strengthen the claims of the current study. The model itself has scope for further improvement, so the algorithm can classify the data while retaining information about damage intensity. In this study, 25 principal components were used because they captured 85% of the variance. After that, each PC had the same variance, yielding a straight line of cumulative variance in the scree plot. However, detailed sensitivity to PC analysis is needed to validate this further. The authors plan to further develop the model by demonstrating its sensitivity to window size across the chosen datasets through a detailed sensitivity analysis. Testing and classifying the thermoset CFRP composite as well as thermoplastic CFRP composites with different layups using the same experimental setup and the above DL-ML framework, respectively, and comparing the results between the two composite types can confirm the hypothesis implied by the paper’s results. Future work employing digital image correlation synchronized with AE acquisition, or controlled tests on specimens with and without fiber waviness, is needed to validate the source of the medium-high frequency AE signals. Furthermore, AE localization using post-mortem CT/microscopy is planned to be explored in future work to validate multiplets. The authors also plan to extend the cross-correlation work by recording a detailed sensitivity study.

## Figures and Tables

**Table 1:** Comprehensive mapping between data-driven features, acoustic emission response parameters, Lamb wave modes, and validated physical damage mechanisms in PAEK/CFRP laminates.

Signal Class	Primary Spectral Band	Dominant Lamb Wave Mode	Acoustic Signal Attributes	Physical Damage Mechanism	Microstructural and Mechanical Validation Evidence
Low Frequency (Class 4)	50–250 kHz	Dominant flexural mode (*A*_0_)	Low peak frequency, long duration (>300 μs), high energy release	Interlaminar delamination	Optical micrographs; flexural wave dominance due to out-of-plane displacement during surface separation; occurs predominantly at high-load stages.
Mid Frequency (Class 3)	250–900 kHz	Dominant extensional mode (*S*_0_) followed by *A*_0_	Moderate peak frequency, medium rise time, low-to-moderate amplitude	Transverse and intralaminar matrix cracking	Optical micrographs; initiated at matrix strain yield threshold (∼0.6–0.8% laminate strain) in 90° and ±45° plies.
Mid-High Frequency (Class 2)	900–1600 kHz	Mixed high-order extensional/flexural modes	High amplitude (>75 dB), burst-type waveform, medium duration	Cooperative multi-fiber breakage in wavy 0° plies	Optical micrograph; localized 0° ply waviness induces combined axial tension and transverse shear, causing simultaneous fracture of adjacent wavy fiber bundles.
High Frequency (Class 1)	1600–3000 kHz	High-order extensional mode (*S*_0_)	Very high frequency, extremely fast rise time (<15 μs), short burst duration	Individual carbon fiber breakage	Dominates near ultimate failure load (>80% UTS); energy release aligns with elastic strain energy release.

## Data Availability

The raw data supporting the conclusions of this article will be made available by the authors on request.
